# NQO1/p65/CXCL12 Axis‐Recruited Tregs Mediate Resistance to Anti‐PD‐1 Plus Lenvatinib Therapy in PIVKA‐II‐Positive Hepatocellular Carcinoma

**DOI:** 10.1002/advs.202511152

**Published:** 2025-09-30

**Authors:** Biao Gao, Yafei Wang, Zhuoya Sun, Haowen Tang, Yinbiao Cao, Hao Jiang, Wenwen Zhang, Yinzhe Xu, Bingyang Hu, Zhe Liu, Guankun Mao, Xuerui Li, Junfeng Li, Tao Wan, Bing Liu, Xiao Zhao, Shunchang Jiao, Chonghui Li, Shichun Lu

**Affiliations:** ^1^ Faculty of Hepato‐Pancreato‐Biliary Surgery Chinese PLA General Hospital Beijing 100853 China; ^2^ Institute of Hepatobiliary Surgery of Chinese PLA Beijing 100853 China; ^3^ Key Laboratory of Digital Hepatobiliary Surgery of Chinese PLA Beijing 100853 China; ^4^ Department of Hepatobiliary Surgery The First Affiliated Hospital of Henan University of Science and Technology Luoyang 471003 China; ^5^ Medical School of Chinese People's Liberation Army Beijing 100853 China; ^6^ Department of Medical Oncology Chinese PLA General Hospital Beijing 100853 China; ^7^ Department of General Surgery Beijing Shijingshan Hospital Beijing 100043 China; ^8^ Department of Hepato‐Pancreato‐Biliary Surgery The Eighth Medical Center of PLA General Hospital Beijing 100091 China

**Keywords:** anti‐PD‐1 plus lenvatinib therapy, CXCL12, Hepatocellular carcinoma, NF‐κB, NQO1, PIVKA‐II, Plerixafor, resistance, Tregs

## Abstract

Immune checkpoint inhibitors (ICIs) combined with anti‐angiogenic agents manifest improved survival in advanced hepatocellular carcinoma (HCC), but responses remain heterogeneous. Although high PIVKA‐II expression correlates with advanced disease stage, early recurrence, shorter survival, and may predict resistance to anti‐PD‐1 plus lenvatinib therapy, the tumor microenvironment (TME) and resistance mechanisms in HCC with high PIVKA‐II expression remain unclear. Clinical data from 156 resected HCC patients and 104 patients treated with anti‐PD‐1 plus lenvatinib are analyzed to correlate PIVKA‐II expression with clinical features and outcomes. Single‐cell RNA sequencing (scRNA‐seq) is performed on tumors from 15 untreated and 7 treated patients. Mechanistic findings are validated in vitro and in vivo. High PIVKA‐II expression is associated with advanced disease stage, increased microvascular invasion (MVI), early recurrence, and poor response to therapy. ScRNA‐seq revealed an immunosuppressive TME enriched with regulatory T cells (Tregs), exhausted CD8⁺ T cells, and SPP1⁺ tumor‐associated macrophages (TAMs). Mechanistically, tumors with high PIVKA‐II expression upregulated NQO1, which stabilized p65 by inhibiting ubiquitination, activating the NF‐κB/CXCL12 axis, and recruiting Tregs. This pathway mediated therapeutic resistance. Plerixafor, a CXCL12 inhibitor, disrupted this axis and significantly enhanced anti‐tumor efficacy when combined with anti‐PD‐1 plus lenvatinib in vivo. PIVKA‐II is a potentially effective biomarker for predicting resistance to anti‐PD‐1 plus lenvatinib therapy. Its high expression denotes an immunosuppressive TME. Targeting the NQO1/CXCL12/Tregs axis with Plerixafor may restore sensitivity and improve outcomes.

## Graphical abstract

High PIVKA‐II expression in HCC correlates with resistance to anti‐PD‐1 plus lenvatinib, driven by an immunosuppressive TME. NQO1/p65/CXCL12 axis recruits Tregs to promote resistance to anti‐PD‐1 plus lenvatinib in HCC with high PIVKA‐II expression. Plerixafor overcomes resistance to anti‐PD‐1 plus lenvatinib in HCC with high PIVKA‐II expression.

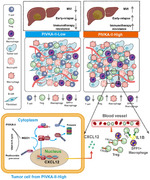

## 1 Introduction

Combination therapy using ICIs and anti‐angiogenic agents has emerged as a first‐line treatment for advanced hepatocellular carcinoma (HCC), offering improved survival and delivering durable responses.^[^ ^1^, ^2^, ^3^ ^]^ Nevertheless, response rates vary widely (23.3%–53.1%), and some patients exhibit primary resistance or hyperprogressive disease.^[^ ^4^, ^5^, ^6^, ^7^ ^]^

The tumor microenvironment (TME)—comprising cancer cells, stromal cells, immune cells, and the extracellular matrix—plays a pivotal role in tumor progression and therapeutic resistance.^[^ ^8^, ^9^, ^10^ ^]^ Single‐cell RNA sequencing (scRNA‐seq) enables high‐resolution profiling of the TME and identifies cell populations and mechanisms involved in immune escape.^[^ ^11^, ^12^, ^13^, ^14^ ^]^

Although protein induced by vitamin K absence or antagonist‐II (PIVKA‐II), a common diagnostic biomarker for HCC,^[^ ^15^ ^]^ is linked to poor prognosis, its role in therapeutic resistance remains underexplored. Prior studies have mainly focused on tumor‐intrinsic aspects,^[^ ^15^, ^16^ ^]^ with limited insight into immune microenvironment alterations in HCC with high PIVKA‐II expression.

This study explores resistance mechanisms to anti‐PD‐1 plus lenvatinib in HCC with high PIVKA‐II expression. Using clinical cohorts, scRNA‐seq, and experimental validation, we identify the NQO1/NF‐κB/CXCL12 axis as a key immunosuppressive pathway that promotes Tregs recruitment and therapeutic resistance. We further demonstrate that blocking CXCL12 can restore therapeutic efficacy.

## 2 Results

### 2.1 HCC with High PIVKA‐II Expression Correlates with Malignant Invasion and Resistance to Anti‐PD‐1 Plus Lenvatinib

To examine the association between PIVKA‐II expression and tumor aggressiveness, clinical data from 156 HCC patients were analyzed, stratified by PIVKA‐II levels^[^ ^17^ ^]^ (> 300 vs. < 300 mAU mL^−1^). Baseline characteristics were balanced between groups (Table S1, Supporting Information).

**Figure 1:**
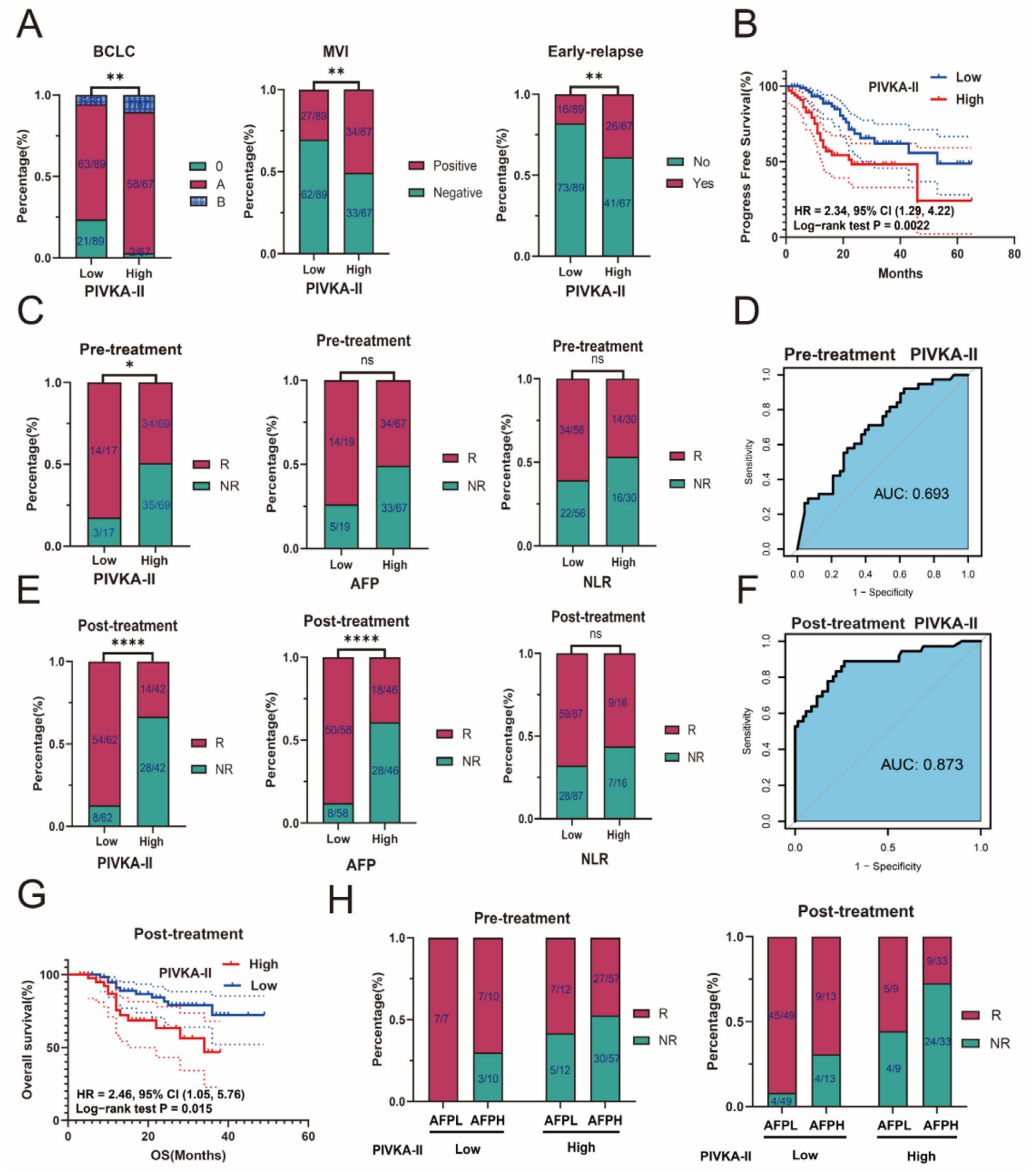
The relationship between PIVKA‐II expression, malignant invasion in HCC, and resistance to anti‐PD‐1 plus lenvatinib therapy. A) Bar chart showing the relationship between high and low PIVKA‐II expression groups and BCLC stage, MVI, and early recurrence. B) Kaplan‐Meier survival curve showing the difference in PFS between the high and low PIVKA‐II expression groups in HCC patients. *P* value was calculated using the log‐rank test. C) Bar chart showing the relationship between treatment response and different PIVKA‐II, AFP, and NLR groupings pre‐treatment. D) ROC curve showing the predictive efficacy of pre‐treatment PIVKA‐II levels in peripheral blood for treatment response. E) Bar chart showing the relationship between treatment response and different PIVKA‐II, AFP, and NLR groupings post‐treatment. F) ROC curve showing the predictive efficacy of post‐treatment PIVKA‐II levels in peripheral blood for treatment response.G) Kaplan‐Meier survival curve showing the difference in OS between the high and low PIVKA‐II expression groups in HCC patients' post‐treatment. *P* value was calculated using the log‐rank test. H) Bar chart showing the relationship between treatment response and AFP levels combined with PIVKA‐II levels pre‐ and post‐treatment. Significance in A,C, and E was analyzed using the Chi‐square test. ^*^ *P* < 0.05, ^**^ *P* < 0.01, ^****^ *P* < 0.0001 and ns, not significant.

Compared with the low PIVKA‐II expression group, high PIVKA‐II expression patients exhibited more aggressive features, including a higher proportion of advanced stage disease (10.4% vs 5.6%), Alpha‐fetoprotein (AFP) positivity (61.2% vs 41.6%), MVI (50.7% vs 30.3%), large tumors (61.3% vs 15.7%), and early recurrence (38.8% vs 18.0%) (**Figures** **1**A; S1A, Supporting Information). Progression‐free survival (PFS) was significantly shorter in the high PIVKA‐II expression group (median: 23 vs 53 months) (Figure 1B).

In an independent cohort of 104 HCC patients treated with anti‐PD‐1 plus lenvatinib, 86 had available pre‐treatment PIVKA‐II measurements (Table S2, Supporting Information). High PIVKA‐II independently predicted poor response (OR: 4.71, 95%CI: 1.17–18.97, P = 0.03) (Table S3, Supporting Information). Objective response rates (ORR) were significantly lower in the high PIVKA‐II expression group (49.3% vs 82.4%) (Figure 1C). By contrast, no significant differences in ORR were observed between groups stratified by AFP (49.3% vs 73.7%) or neutrophil‐to‐lyymphocyte ratio (NLR) (46.7% vs 60.7%) (Figure 1C). Pre‐treatment peripheral blood PIVKA‐II levels better predicted treatment response (AUC = 0.693) than AFP (AUC = 0.578) or NLR (AUC = 0.568) (Figures 1D; S1B, Supporting Information). These results indicate that patients with high PIVKA‐II expression are more likely to be resistant to anti‐PD‐1 plus lenvatinib, and pre‐treatment peripheral blood PIVKA‐II levels may serve as a crucial indicator for predicting the efficacy of anti‐PD‐1 plus lenvatinib therapy in HCC.

An analysis of the clinical data from 104 HCC patients with post‐treatment PIVKA‐II measurements revealed that there were no significant differences in baseline characteristics between the two groups (Table S4, Supporting Information). Logistic regression identified high post‐treatment PIVKA‐II l (OR: 9.68, 95% CI: 2.90‐32.31, P<0.001) and high AFP (OR: 4.69, 95% CI: 1.27‐15.19, P = 0.019) were independent risk factors affecting the efficacy of anti‐PD‐1 plus lenvatinib therapy (Table S5, Supporting Information). Compared with the low PIVKA‐II expression group (*n* = 62), the high PIVKA‐II expression group (*n* = 42) showed markedly lower ORR (33.3% vs 87.1%) (Figure 1E). Similarly, the high‐AFP group (39.1% vs 86.2%) had a poorer response, whereas NLR strata did not differ significantly (56.3% vs 67.8%) (Figure 1E). Receiver Operating Characteristic (ROC) curve analysis indicated that post‐treatment PIVKA‐II expression had a strong ability to predict treatment response (AUC = 0.873) (Figure 1F), outperforming AFP (AUC = 0.790) and NLR (AUC = 0.490) (Figure S1C, Supporting Information). Kaplan‐Meier analysis further demonstrated worse long‐term survival in the high‐PIVKA‐II group (HR = 2.75, 95% CI (1.8, 6.38), P = 0.0187) (Figure 1G), indicating that post‐treatment PIVKA‐II levels not only predict treatment response but also serve as a prognostic indicator.

Although AFP showed lower discriminative performance than PIVKA‐II, it remained an independent predictor. Patients with low AFP and PIVKA‐II had the best responses; those with high levels of both had the worst. Intermediate response was seen in discordant expression groups (Figure 1H), suggesting differing mechanisms of therapy resistance. Combined AFP and PIVKA‐II testing may therefore improve personalized treatment and risk stratification.

### 2.2 scRNA‐Seq Reveals the Heterogeneity of TME Between the Low‐ and High‐Expression PIVKA‐II Groups in HCC

**Figure 2:**
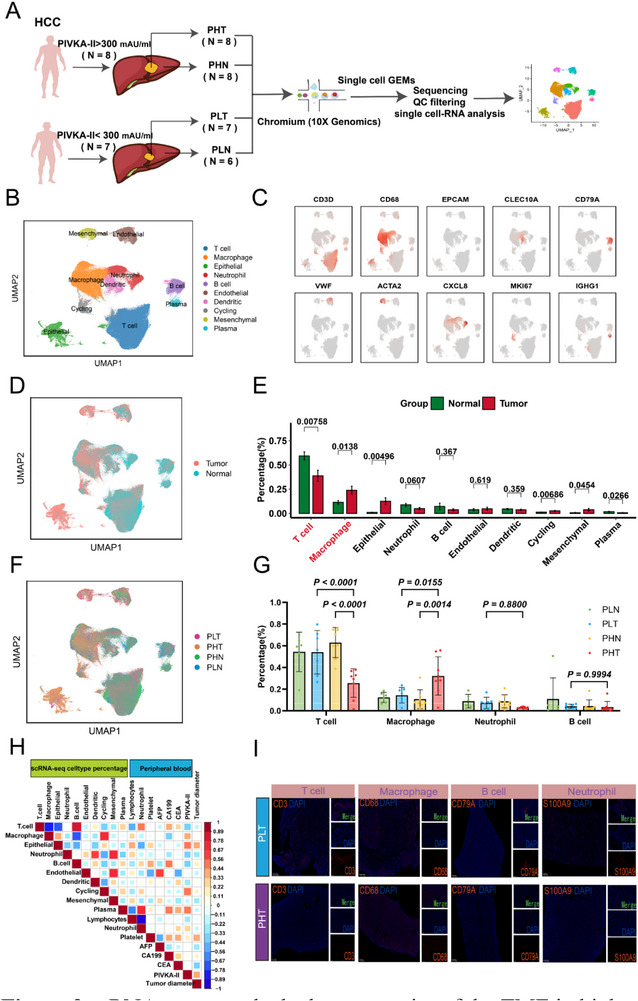
scRNA‐seq reveals the heterogeneity of the TME in high and low PIVKA‐II expression groups. A) Flowchart of scRNA‐seq performed on 15 primary HCC patients. B) UMAP plot showing the distribution of annotated cell types. C) UMAP plot showing the expression distribution of characteristic genes in cells. D) UMAP plot showing the distribution of cells derived from tumor tissue and adjacent normal tissue. E) Bar chart showing the differences in the proportion of different cell types between tumor tissue (*n* = 15) and adjacent normal tissue (*n* = 14). F) UMAP plot showing the distribution of cells from PLN, PLT, PHN, and PHT. PHN: high PIVKA‐II expression group, adjacent normal tissue; PLN: low PIVKA‐II expression group, adjacent normal tissue. G) Bar chart showing the differences in the proportions of T cells, macrophages, neutrophils, and B cells among PLN (*n* = 6), PLT (*n* = 7), PHT (*n* = 8), and PHN (*n* = 8). H) Correlation heatmap showing the relationship between the proportion of different cell types in tumor tissue and clinical indicators. I) mIHC showing differences in T cell, macrophage, B cell, and neutrophil infiltration between PLT and PHT. Significance in E and G was analyzed using the two‐sided Student's *t*‐test. Data are presented as mean ±SD. Each dot corresponds to one sample.

ScRNA‐seq of 29 tumor/adjacent‐tissue samples from 15 untreated HCC patients (**Figure** **2**A; Table S6, Supporting Information) yielded 304,937 high‐quality cells(Figure S2A–C, Table S7, Supporting Information), with 13 cell types annotated (Figures 2B,C; S2D–G, Tables S8 and S9, Supporting Information). T cell, neutrophil, and plasma cell infiltration was reduced in tumor tissue (Figure 2D,E); whereas macrophage and stromal cell increased (Figure 2D,E), consistent with an immune‐cold tumor microenvironment.

Further analysis revealed pronounced differences in immune infiltration between high and low PIVKA‐II expression groups (Figure 2F). Anti‐tumor immune populations—including cytotoxic T cells, neutrophils, B cells, and dendritic cells (DCs)—were predominantly enriched in the low PIVKA‐II expression group. By contrast, immunosuppressive macrophages and cycling cells were markedly elevated in the high PIVKA‐II expression group (Figures 2G; S2H,I, Supporting Information).

We also found that serum PIVKA‐II levels were correlated positively with the infiltration of epithelial cells, macrophages, and plasma cells, and negatively correlated with the infiltration of T cells, neutrophils, B cells, and dendritic cells (Figure 2H). Multiplex immunohistochemistry (mIHC) confirmed reduced immune cell infiltration and increased macrophages in high PIVKA‐II expression group tumors (Figure 2I), supporting an immunosuppressive TME.

### 2.3 High PIVKA‐II Expression Tumors Exhibited Increased Infiltration of Immunosuppressive T Cells Within the TME

**Figure 3:**
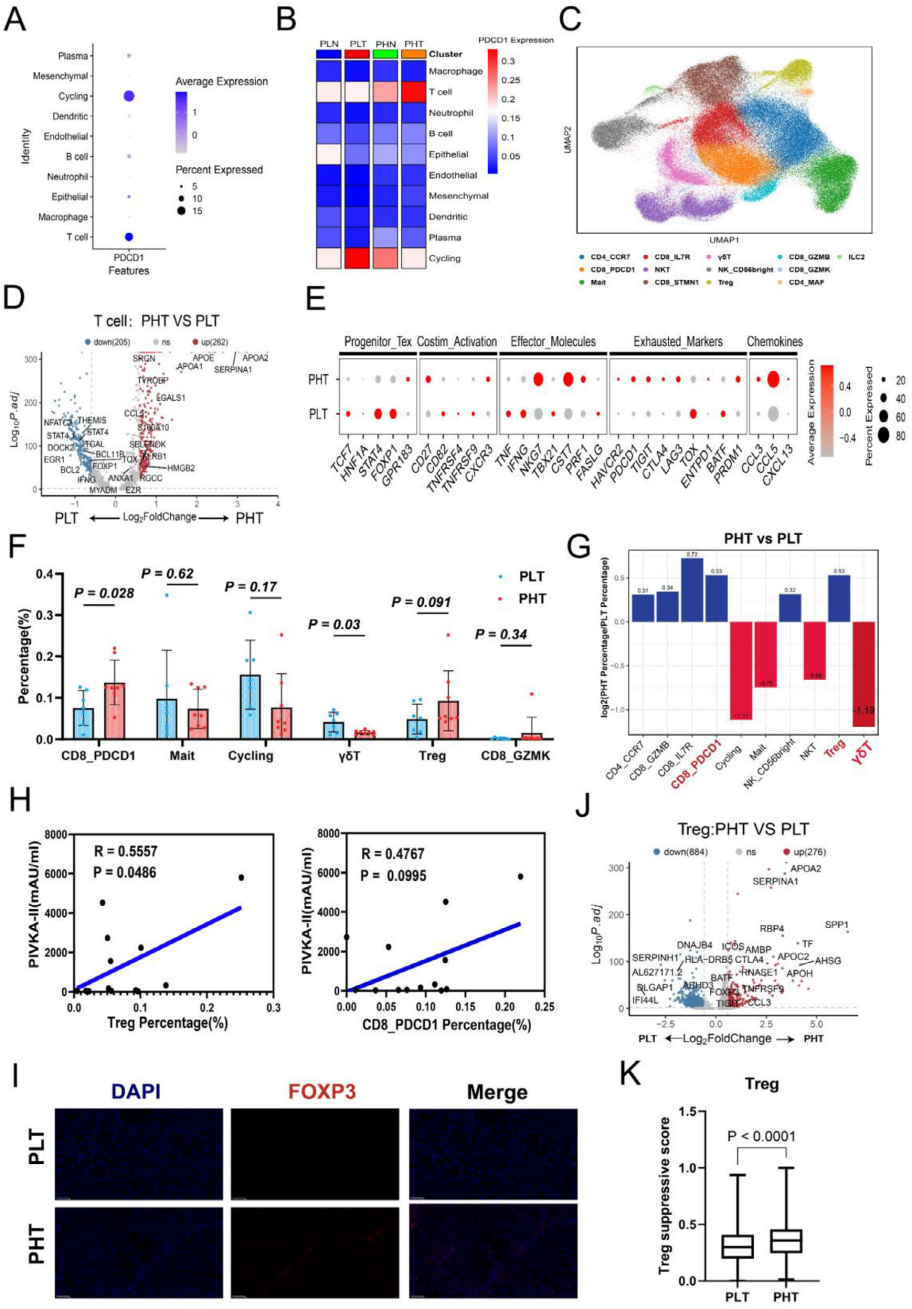
Increased infiltration of immunosuppressive T cells in tumor tissue of the high PIVKA‐II expression group. A) Bubble chart showing the expression of PDCD1 in different cell types. B) Heatmap showing the expression of PDCD1 in different groups and cell types. C) UMAP plot showing the distribution of T cell subgroups. D) Volcano plot showing differentially expressed genes in T cells from the two groups. E) Bubble chart displaying functionally relevant genes enriched in PLT and PHT.F) Bar chart illustrating the differences in the proportions of T cell subgroups between PLT (*n* = 7) and PHT (*n* = 8).G) Bar chart showing the fold changes in T cell subgroup infiltration ratios between PHT and PLT.H) Correlation scatter plot showing the relationship between the proportions of Tregs and CD8_PDCD1 cells and the levels of PIVKA‐II in peripheral blood. I) mIHC showing the differences in Tregs cell infiltration between PLT and PHT. J) Volcano plot showing differentially expressed genes in Tregs cells from the two groups. K) Boxplot showing the differences in immunosuppressive scores of Treg cells between PHT (*n* = 1911) and PLT (*n* = 1652). Significance in F was analyzed using the two‐sided Student's *t*‐test. Data are presented as mean ±SD. Each dot corresponds to one sample. Significance in K was analyzed using the two‐sided Wilcoxon rank‐sum test. Center line: median; box edges: 25th/75th percentiles; whiskers: 1.5*IQR; upper and lower bars: 95% CI.

T cells showed the most pronounced differences between the PLT and PHT (Figure 2G). PD‐1 expression, predominantly in T cells (**Figure** **3**A), was highest in PHT (Figure 3B), suggesting a potential role in treatment response. From 149,043 T/NK compartment, 13 subtypes were identified via dimensionality reduction (Figures 3C; S3A, Supporting Information), including naive CD4 T cells (CD4_CCR7), exhausted CD8 T cells (CD8_PDCD1), memory CD8 T cells (CD8_IL7R), NKT cells (NKT), cycling T cells (Cycling), γδT cells (γδT), NK cells (NK_CD56bright), Tregs, tissue‐resident CD8 T cells (CD8_GZMB), effector memory CD8 T cells (CD8_GZMK), follicular helper T cells (CD4_MAF), mucosal‐associated invariant T cells (Mait), and type II innate lymphoid cells (ILC2) (Figure 3C).

Immune cytotoxic genes were upregulated in the low PIVKA‐II expression group, while immunosuppressive genes were upregulated in the high PIVKA‐II expression group (Figure 3D,E). Pathways related to T cell activation and cytotoxicity were enriched in the low PIVKA‐II expression (Figure S3B, Supporting Information). Conversely, T cells from the high PIVKA‐II expression group exhibited higher exhaustion scores (Figure S3C, Supporting Information), indicating an immunosuppressive state, likely due to increased infiltration of immunosuppressive T cells. Subtype distribution also differed significantly (Figures 3F; S3D, Supporting Information), with γδT cells significantly reduced in PHT (Figure 3F,G), a group linked to immune activation (Figure S3E, Supporting Information). Consistently, higher γδT cell scores in TCGA‐LIHC and GSE14520 datasets correlated with better prognosis (Figure S3F,G, Supporting Information).

Notably, CD8_PDCD1 and Treg cells were significantly more abundant in tumors from the high PIVKA‐II expression group(Figure 3F,G), with infiltration levels correlating positively with serum PIVKA‐II levels (Figure 3H). mIHC confirmed increased FOXP3^+^ Treg cells in the high PIVKA‐II expression group (Figure 3I). Transcriptome data showed upregulation of both immunosuppressive and activation genes (Figure 3J). Furthermore, Treg cells derived from the high PIVKA‐II expression group exhibited higher immunosuppressive scores (Figure 3K), consistent with functional activation.

Cell communication analysis revealed more diverse and stronger interactions between tumor and Treg cells in PHT (Figure S3H, Supporting Information). NicheNet indicated that tumor‐derived chemokines (CCL5, TGFB1) interacted with Tregs receptors (CCR4, CCR5, CXCR3), promoting the Treg recruitment (Figure S3I, Supporting Information). This indicates PIVKA‐II‐positive tumor cells facilitate Tregs recruitment, reinforcing an immunosuppressive TME, associated with poorer prognosis and shorter PFS.

### 2.4 Increased Infiltration of Angiogenesis‐Promoting SPP1^+^TAM in the High PIVKA‐II Expression Group HCC

In PHT, macrophages are the most enriched immune cell type (Figure 2G) and exhibit the most significant proportion changes (Figure S4A, Supporting Information). Overexpression of macrophage‐related genes, such as SPP1, CD9, and CSF1R, in PHT (Figure S5A,B, Supporting Information) indicates their crucial role in the TME of high PIVKA‐II expression tumors. We classified macrophages into seven subgroups based on marker genes (Figures S4B and S5C, Supporting Information), finding that SPP1^+^ TAMs promote angiogenesis by upregulating cytokines (e.g., IL1B) and chemokines (e.g., CXCL8, CXCL3, and CXCL2) and angiogenesis‐related genes (e.g., VEGFA, EREG, and AREG) (Figures S4C and S5D, Supporting Information).

In the high PIVKA‐II expression group, SPP1^+^ TAMs infiltration was significantly increased (Figure S4D, Supporting Information), while other subgroups manifested no significant changes. SPP1^+^ TAMs are involved in pathways related to hypoxia, angiogenesis, and inflammation (Figure S4E, Supporting Information), potentially promoting angiogenesis and the formation of a hypoxic microenvironment. In adjacent non‐tumor tissue, SPP1^+^ TAMs exhibit an M1 phenotype, while in tumor tissue, particularly in PHT, SPP1^+^ TAMs exhibit an M2 phenotype (Figure S4F, Table S10, Supporting Information), displaying stronger immunosuppressive features.

Furthermore, SPP1^+^ TAMs in PHT demonstrate enhanced angiogenic ability and inhibit lymphocyte migration (Figure S5E, Table S10, Supporting Information), supporting their role in promoting tumor progression. In the TCGA‐LIHC and GSE14520 HCC cohorts, M2‐polarized SPP1+ TAMs are associated with poor prognosis (Figure S5F, Supporting Information). Cell communication analysis revealed significantly enhanced interactions between SPP1^+^ TAMs and endothelial cells in PHT (Figure S5G,H, Supporting Information), with SPP1 specifically enriched in PHT (Figure S5I, Supporting Information). This indicates the crucial role of SPP1^+^ TAMs in cellular interactions. The analysis also revealed that SPP1^+^ TAMs interact with endothelial cells via the SPP1‐(ITGAV+ITGB1)/(ITGA5+ITGB1) axis (Figure S5J, Supporting Information), promoting aberrant angiogenesis, a finding that coheres with prior studies. Collectively, these findings suggest that SPP1^+^ TAMs exacerbate hypoxia and drive tumor progression in high PIVKA‐II expression tumors.

### 2.5 The Interaction Between Treg cells and SPP1^+^ TAMs may Contribute to the Immunosuppressive Microenvironment in the High PIVKA‐II Expression Group HCC

We observed highly similar infiltration patterns of Treg cells and SPP1^+^ TAMs in PHT (Figure S6A, Supporting Information), indicating a potential interaction between them, and spatial transcriptomics analysis revealed their colocalization (Figure S6B, Supporting Information). This indicates that SPP1^+^ TAMs and Treg cells in PHT may jointly promote an immunosuppressive microenvironment. We classified 15 HCC patients into four groups based on the infiltration levels of SPP1^+^ TAMs and Treg cells. The results indicated that the SPP1^+^TAM^high^_Treg^high^ group exhibited increased CD8_PDCD1 infiltration, whereas the SPP1^+^TAM^low^_Treg^low^ group manifested reduced infiltration (Figure S6C, Supporting Information). Consistently, the TCGA database displayed similar trends for immune checkpoint gene expression (Figure S6D, Supporting Information), indicating a synergistic role of Treg cells and SPP1^+^ TAMs. NicheNet analysis indicated that SPP1^+^ TAMs promote Treg cell recruitment and immunosuppression in the TME by secreting IL18, IL1B, and CXCL12, which interact with receptors on Tregs (Figure S6E, Supporting Information). These results suggest that Treg cells and SPP1^+^ TAM interactions synergistically support the immunosuppressive environment in high PIVKA‐II expression tumors.

### 2.6 Tregs Infiltration Contributes to Resistance to anti‐PD‐1 Plus Lenvatinib Therapy in Patients with High PIVKA‐II Expression

**Figure 4:**
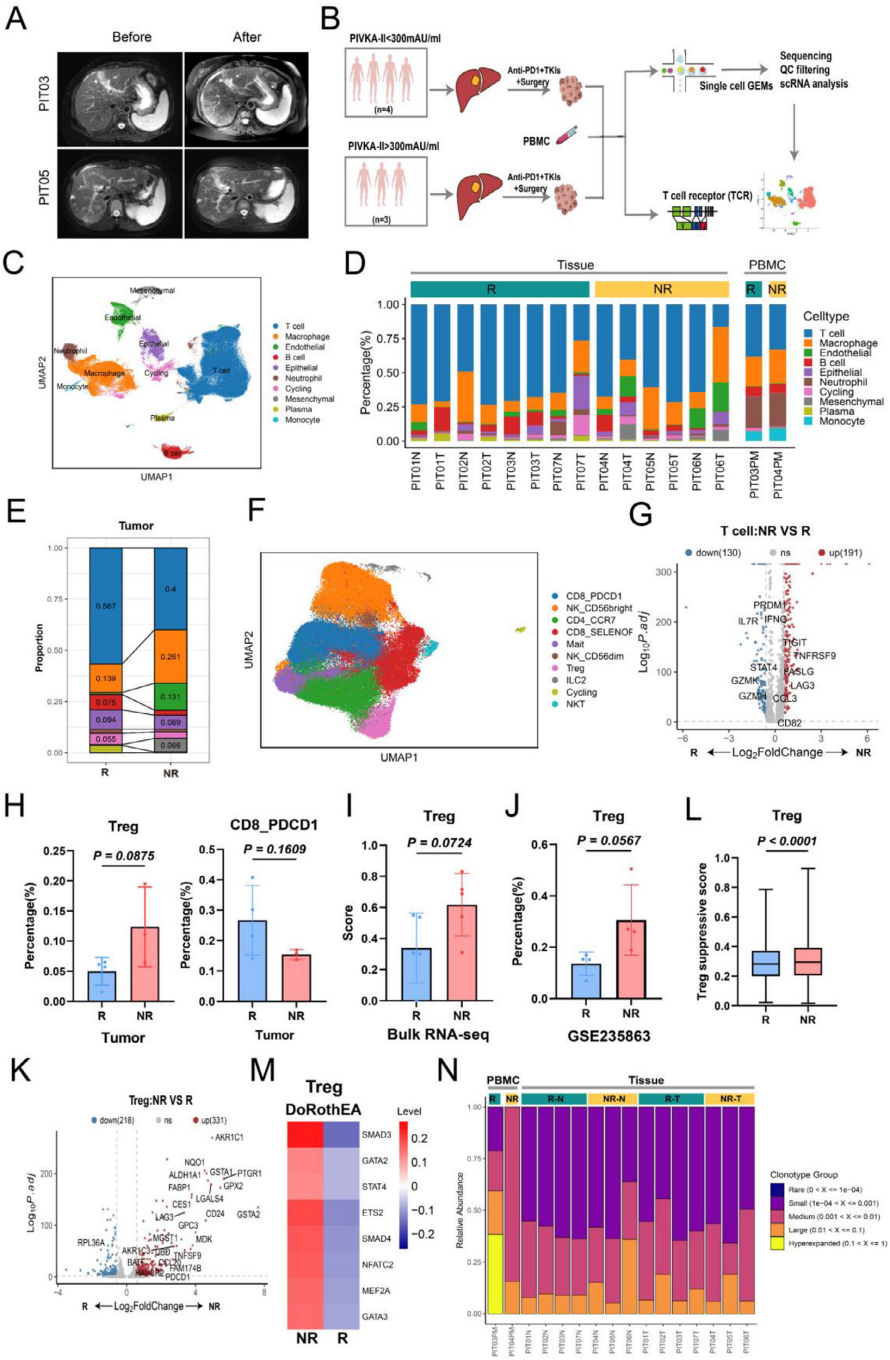
Treg cell infiltration leads to resistance to anti‐PD‐1 plus lenvatinib therapy in patients with high PIVKA‐II expression. A) Typical magnetic resonance imaging (MRI) results showing tumor changes pre‐ and post‐treatment in patients PIT03 and PIT05. B) Flowchart of scRNA‐seq on tumor and adjacent normal tissue from 7 HCC patients receiving anti‐PD‐1 plus lenvatinib therapy. C) UMAP plot showing the distribution of all cell types. D) Stacked bar chart displaying the cell composition from different patients and tissue sources. E) Stacked bar chart showing differences in cell composition between PHT and PLT in tumor tissue. F) UMAP plot showing the distribution of different T cell subgroups. G) Volcano plot showing differentially expressed genes in T cells between the NR and R groups. H) Bar chart showing the differences in the proportions of Treg and CD8_PDCD1 cells between the NR (*n* = 3) and R (*n* = 4) groups. I) Bar chart showing the differences in Treg cell scores between the NR group (*n* = 5) and R group (*n* = 5) in the bulk RNA sequencing cohort of 10 HCC patients receiving anti‐PD‐1 plus lenvatinib therapy. J) Bar chart showing the differences in the proportion of Treg cells between the NR group (*n* = 4) and R group (*n* = 4) in the GSE235863 cohort. K) Volcano plot showing differentially expressed genes in Treg cells between the NR and R groups. L) Boxplot showing the differences in immunosuppressive scores of Treg cells between the NR (*n* = 1386) and R (*n* = 1527) groups. M) Heatmap showing the differences in transcription factor regulation of Treg cells between the NR and R groups. N) Bar chart showing the proportions of TCR clonotypes in different samples. TCR: T‐cell receptor. Significance in H,I and J was analyzed using the two‐sided Student's *t*‐test.Data are presented as mean ±SD. Each dot corresponds to one sample. Significance in L was analyzed using the two‐sided Wilcoxon rank‐sum test. Center line: median; box edges: 25th/75th percentiles; whiskers: 1.5*IQR; upper and lower bars: 95% CI.

In patients receiving anti‐PD‐1 plus lenvatinib therapy, the high PIVKA‐II expression group showed poorer treatment responses (Figures 4A‐B; Table S11, Supporting Information), with decreased infiltration of immune cytotoxic cells and increased presence of immunosuppressive macrophages and endothelial cells (Figures 4C–E; S7A–G, Table S12, Supporting Information). Treg cells were particularly abundant in non‐responder (NR) patients, while cytotoxic CD8+ cells (CD8_PDCD1) were reduced (Figures 4F–H; S7H,I, Supporting Information). Multiple external datasets corroborated higher Treg cells enrichment in the resistance group (Figure 4I,J, Supporting Information). Transcriptomic analysis demonstrated the upregulation of immunosuppressive and proliferative genes in the Treg cells from NR patients (Figures 4K,L; S7J, Supporting Information), further supporting their role in therapy resistance. These results indicate that increased Treg infiltration is a critical factor in resistance to anti‐PD‐1 plus lenvatinib therapy in patients with high PIVKA‐II expression. Transcription factor analysis indicated that Treg cells in the NR group were regulated by multiple transcription factors (such as SMAD3, ETS2, NFATC2, and STAT4), which play key roles in activation and proliferation (Figure 4M). Moreover, T cells in the responder(R) group showed more clonal expansion, while clonal expansion was restricted in the NR group (Figure 4N).

### 2.7 Tumor Cells in the High PIVKA‐II Expression Group Upregulate NQO1 Mediated Anti‐PD‐1 Plus Lenvatinib Therapy Resistance

**Figure 5:**
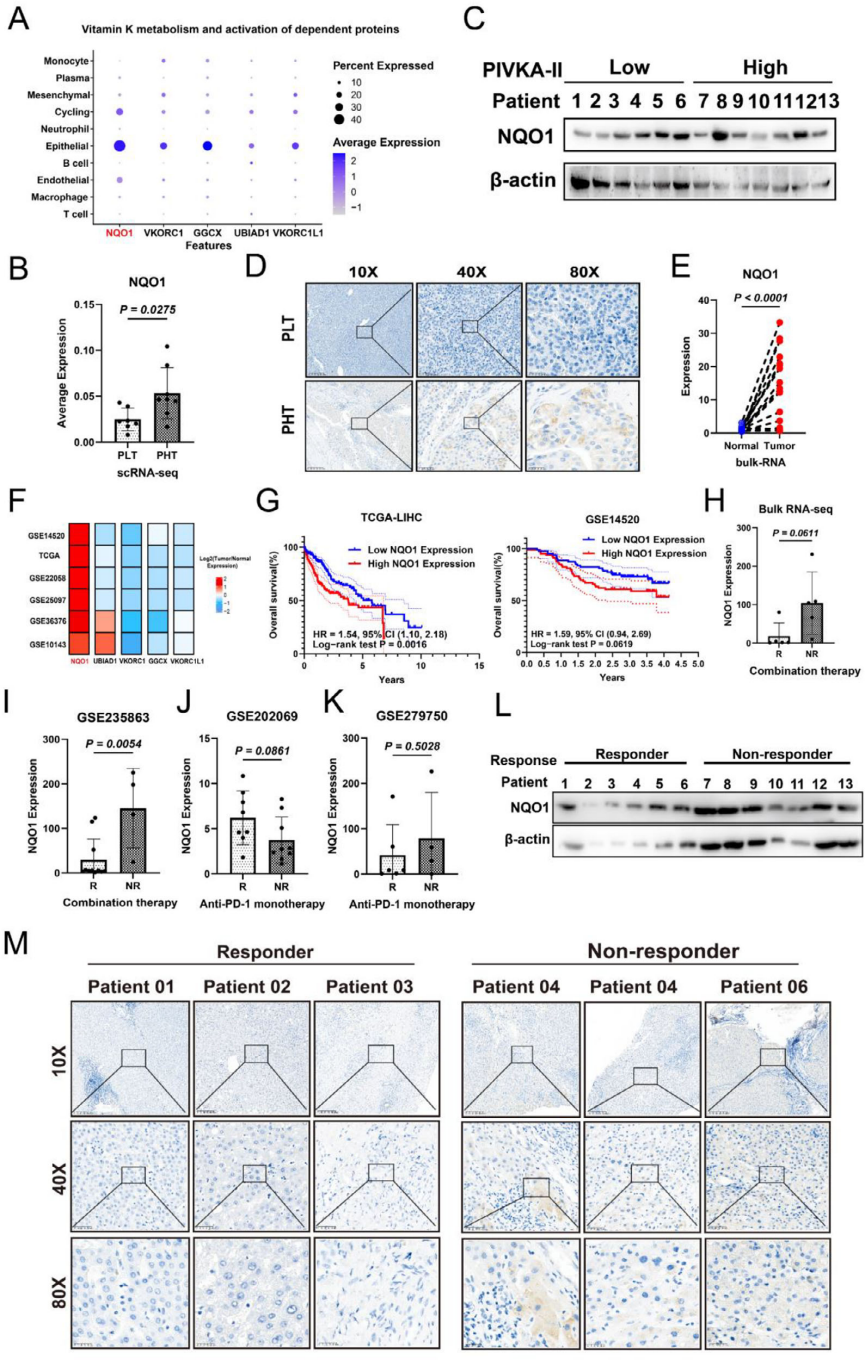
NQO1 is upregulated in tumors with high PIVKA‐II expression and is associated with resistance to anti‐PD‐1 antibody plus lenvatinib. A) Bubble chart showing the expression of key genes in the vitamin K cycle pathway across different cell types. B) Bar chart showing the differences in NQO1 expression in PLT (*n* = 7) and PHT (*n* = 8) tumor tissue from scRNA‐seq data of 15 HCC patients. C) Western blot results showing NQO1 protein expression in the high and low PIVKA‐II expression groups. D) IHC results showing NQO1 protein expression in the high and low PIVKA‐II expression groups. E) Scatter plot showing the differences in NQO1 expression between tumor (*n* = 17) and adjacent normal tissue (*n* = 17) in bulk RNA sequencing data from 17 HCC patients. F) Heatmap showing the fold difference in the expression of key genes in the vitamin K cycle pathway between tumor and adjacent normal tissue across different HCC datasets. G) Kaplan‐Meier survival curve showing the relationship between NQO1 expression levels and OS in TCGA and GSE14520 datasets. *P* value was calculated using the log‐rank test. H) Bar chart showing the difference in NQO1 expression levels between the NR (*n* = 5) and R group (*n* = 5) in the bulk RNA sequencing data from 10 HCC patients receiving anti‐PD‐1 plus lenvatinib therapy. I) Bar chart showing the difference in NQO1 expression levels between the NR (*n* = 4) and R group (*n* = 11) in the GSE235863 dataset of 15 HCC patients receiving anti‐PD‐1 plus lenvatinib therapy. J) Bar chart showing the difference in NQO1 expression levels between the NR (*n* = 9) and R group (*n* = 8) in the GSE202069 dataset of 17 HCC patients receiving anti‐PD‐1 monotherapy. K) Bar chart showing the difference in NQO1 expression levels between the NR (*n* = 4) and R group (*n* = 6) in the GSE279750 dataset of 10 HCC patients receiving anti‐PD‐1 monotherapy. L) Western blot results confirming the increased NQO1 protein levels in the NR group of anti‐PD‐1 plus lenvatinib therapy. M) IHC results showing the increased NQO1 protein levels in the NR group of anti‐PD‐1 plus lenvatinib therapy. Significance in E was analyzed using the paired t‐test. Significance in B,H,I,J, and K was analyzed using the two‐sided Student's *t‐*test. Data are presented as mean ±SD. Each dot corresponds to one sample.

HCC cells are the primary source of PIVKA‐II, a protein aberrantly generated during impaired vitamin K‐dependent carboxylation of prothrombin. Disruption of the vitamin K cycle (e.g., by deficiency or VKOR inhibition) promotes PIVKA‐II synthesis (Figure S8A, Supporting Information). ScRNA‐seq data show that vitamin K cycle genes(NQO1, UBIAD1, GGCX, VKORC1, VKORC1L1) are specifically expressed in tumor cells (**Figure** **5**A). Notably, only NQO1 was significantly upregulated in high PIVKA‐II expression HCC cohorts (TCGA‐LIHC; Figure S8B, Supporting Information) and PHT groups (scRNA‐seq; Figure 5B). Consistently, elevated NQO1 protein levels were validated in high PIVKA‐II expression patient tissue (Figures 5C,D; S8C, Supporting Information). This implicates NQO1 upregulation in HCC‐driven PIVKA‐II generation.

Bulk RNA‐seq of paired tumor/normal tissue (n = 17) and external datasets confirmed significant NQO1 overexpression in tumor tissue(Figure 5E,F), associating with poor prognosis (Figure 5G). Critically, NQO1 expression was markedly higher in non‐responders to anti‐PD‐1 plus lenvatinib (Figure 5H,I), but not to anti‐PD‐1 monotherapy (Figure 5J,K). Western blot and IHC confirmed elevated NQO1 protein in NR tumors (Figure 5L,M).

### 2.8 NQO1 Promotes Treg Cell Recruitment by Upregulating CXCL12

**Figure 6:**
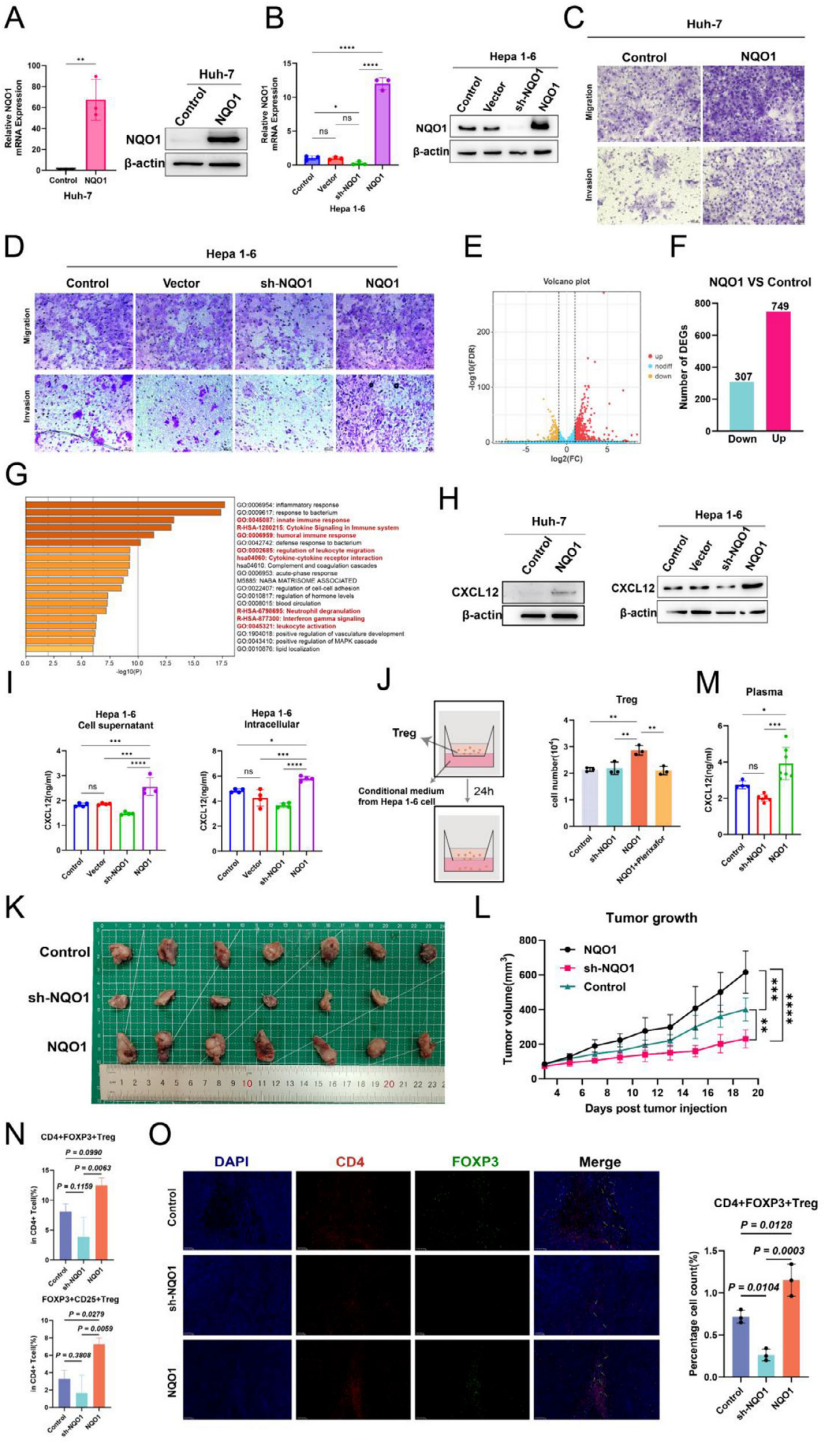
NQO1 promotes Treg cell infiltration by upregulating CXCL12 in HCC. A) qPCR and WB results showing the mRNA and protein expression of NQO1 in the control (*n* = 3) and NQO1‐overexpressing (*n* = 3) group Huh‐7 cells. B) qPCR and WB results showing the mRNA and protein expression of NQO1 in the Control (*n* = 3), Vector (*n* = 3), sh‐NQO1 (*n* = 3), and NQO1 (*n* = 3) group Hepa 1‐6 cells. C) Transwell experiment showing the differences in migration and invasion ability between the Control and NQO1‐overexpressing group Huh‐7 cells. D) Transwell experiment showing the differences in migration and invasion ability between the Control, Vector, sh‐NQO1, and NQO1 group Hepa 1‐6 cells. E) Volcano plot showing differentially expressed genes between the control and NQO1‐overexpressing group Huh‐7 cells. F) Bar chart showing the number of upregulated and downregulated genes in NQO1‐overexpressing group Huh‐7 cells. G) Bar chart displaying the signaling pathways involved in the upregulated genes in the NQO1‐overexpressing group Huh‐7 cells. H) Western blot results showing CXCL12 expression in Huh‐7 and Hepa 1‐6 cells from different treatment groups. I) ELISA results showing the concentrations of CXCL12 protein in the supernatant and intracellular fractions of Control (*n* = 4), Vector (*n* = 4), sh‐NQO1 (*n* = 4), and NQO1 (*n* = 4) groups of Hepa 1‐6 cells. J) Transwell experiment showing the differences in Treg cell recruitment ability induced by the supernatant from Control (*n* = 3), Vector (*n* = 3), sh‐NQO1 (*n* = 3), and NQO1 (*n* = 3) group Hepa 1‐6 cells. K) Tumor specimens from Control, sh‐NQO1, and NQO1 groups of tumor‐bearing mice. L) Line graph showing changes in tumor volume over time in tumor‐bearing mice from the Control(*n* =7), sh‐NQO1(*n* = 6), and NQO1 (*n* = 7) groups. M) ELISA results showing the levels of CXCL12 in the peripheral blood of mice from Control (*n* = 4), sh‐NQO1 (*n* = 6), and NQO1 (*n* = 7) groups. N) Flow cytometry analysis showing the proportion of CD4^+^FOXP3^+^Treg and CD4^+^FOXP3^+^CD25^+^Treg cells in tumor tissue of mice from Control (*n* = 4), sh‐NQO1 (*n* = 4), and NQO1 (*n* = 4) groups. O) mIHC showing the differences in CD4^+^FOXP3^+^Treg cell infiltration between Control (*n* = 3), sh‐NQO1 (*n* = 3), and NQO1 (*n* = 3) groups. Significance in A,B,I,J,M,L,N, and O was analyzed using the two‐sided Student's *t*‐test. Data are presented as mean ±SD. Each dot corresponds to one sample. ^*^ *P* < 0.05, ^**^ *P* < 0.01, ^***^ *P* < 0.001, ^****^ *P* < 0.0001 and ns, not significant.

To investigate the function of NQO1 in HCC, we constructed NQO1 overexpression and knockout mouse and human HCC cell lines, and confirmed the changes in NQO1 expression through qPCR and Western blot analysis (**Figure** **6**A,B). Wound healing assays indicated that NQO1‐overexpressing Huh‐7 cells exhibited enhanced migratory ability (Figure S9A, Supporting Information). In Hepa 1‐6 cells, the migration ability was significantly reduced in the NQO1 knockout group(sh‐NQO1), while it was significantly increased in the NQO1‐overexpressing group (Figure S9B, Supporting Information). Further Transwell assays revealed that NQO1‐overexpressing Huh‐7 cells increased invasive capacity (Figure 6C), while the invasiveness of sh‐NQO1 Hepa 1‐6 cells was decreased (Figure 6D). Overall, these results indicate that the upregulation of NQO1 promotes the migration and invasion abilities of HCC cells.

To explore the impact of NQO1 on HCC cells, we performed bulk RNA sequencing on the control and NQO1‐overexpressing group Huh‐7 cells. The results indicated that NQO1 was significantly upregulated in the NQO1‐overexpressing Huh‐7 cells (Figure S9C, Supporting Information), and differential analysis identified 749 genes that were upregulated in the NQO1‐overexpressing group (Figure 6E,F). Enrichment analysis revealed that these genes were primarily related to immune responses and cytokine signaling pathways (Figure 6G), with CXCL12 being notably upregulated in the NQO1‐overexpressing group (Figure S9E,F, Supporting Information). CXCL12, which regulates the recruitment and function of Treg cells, may be influenced by NQO1. Further experimental validation indicated that in NQO1‐overexpressing Huh‐7 and Hepa 1‐6 cells, both the protein (Figure 6H) and mRNA (Figure S9G, Supporting Information) levels of CXCL12 were significantly increased, while the sh‐NQO1 group exhibited a significant decrease (Figures 6H; S9G, Supporting Information). ELISA results also confirmed that the concentration of CXCL12 was significantly elevated in cell lysates and culture supernatants from the NQO1‐overexpressing group (Figure 6I). Overall, these results indicate that the upregulation of NQO1 promotes the expression of CXCL12, which may affect the TME by regulating immune cell migration and function.

Studies have revealed that CXCL12 plays a key role in promoting Treg cell recruitment in both inflammation and the TME. To investigate this, we collected the supernatants from Hepa 1‐6 cells under different treatment conditions and performed a co‐culture experiment with mouse Treg cells using Transwell plates. The experimental results demonstrated that the supernatant from sh‐NQO1 Hepa 1‐6 cells significantly decreased the number of Treg cells recruited, while the supernatant from NQO1‐overexpressing Hepa 1‐6 cells significantly increased Treg cell recruitment (Figure 6J). After using the CXCR4 antagonist Plerixafor to inhibit the CXCL12/CXCR4 axis, the Treg cell recruitment effect of NQO1‐overexpressing Hepa 1‐6 cells was significantly decreased (Figure 6J), further confirming the critical role of CXCL12. Notably, PIVKA‐II levels were significantly downregulated in sh‐NQO1 Hepa 1‐6 cells (Figure S9D, Supporting Information), indicating a functional association between NQO1 expression and PIVKA‐II production.

In the mouse tumor model, the NQO1‐overexpressing group exhibited faster tumor growth and larger tumor volumes (Figure 6K,L), with significantly elevated levels of CXCL12 in peripheral blood (Figure 6M). Flow cytometry analysis and mIHC further confirmed that the infiltration of Treg cells in the tumor tissue was significantly increased in the NQO1‐overexpressing group, while the infiltration of Treg cells was significantly reduced in the sh‐NQO1 group (Figures 6N,O; S10A, Supporting Information). These results indicate that NQO1, by promoting the expression of CXCL12, enhances Treg cell recruitment. Thus, the formation of an immunosuppressive TME is fostered, and tumor progression is promoted.

### 2.9 NQO1 Regulates the Expression of CXCL12 by Inhibiting the Ubiquitination of p65 and Activating the NF‐κB Signaling Pathway

**Figure 7:**
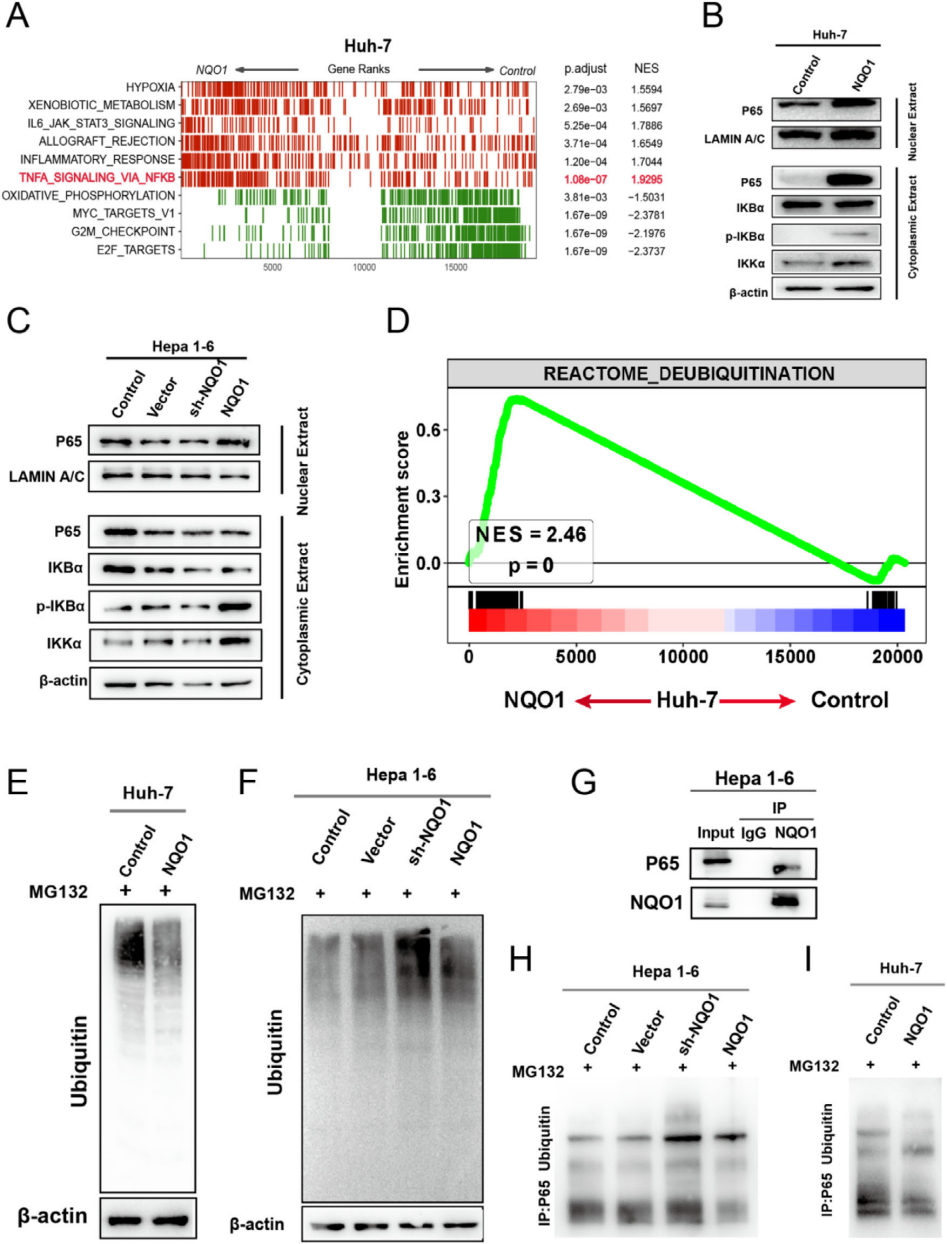
NQO1 activates the NF‐κB signaling pathway by inhibiting p65 ubiquitination. A) Heatmap showing the enriched signaling pathways in the control and NQO1‐overexpressing group Huh‐7 cells. B) Western blot results showing the expression of key proteins in the NF‐κB signaling pathway in the control and NQO1‐overexpressing group Huh‐7 cells. C) Western blot results showing the expression of key proteins in the NF‐κB signaling pathway in Control, Vector, sh‐NQO1, and NQO1 groups Hepa 1‐6 cells. D) GSEA results showing the enrichment of the deubiquitination signaling pathway in the control and NQO1‐overexpressing group Huh‐7 cells. E) Western blot results showing the ubiquitination levels of proteins in the control and NQO1‐overexpressing group Huh‐7 cells. F) Western blot results showing the ubiquitination levels of proteins in Control, Vector, sh‐NQO1, and NQO1 groups Hepa 1‐6 cells. G) Co‐immunoprecipitation (Co‐IP) results showing that NQO1 can bind to p65. H) Western blot results showing the ubiquitination levels of p65 protein in Control, Vector, sh‐NQO1, and NQO1 groups Hepa 1‐6 cells. I) Western blot results showing the ubiquitination levels of p65 protein in the control and NQO1‐overexpressing group Huh‐7 cells.

The preceding analysis indicates that NQO1 promotes Treg cell recruitment in HCC cells by regulating the expression of CXCL12. We further explored the specific mechanism by which NQO1 regulates CXCL12 expression. GSEA of bulk RNA‐seq data from NQO1‐overexpressing and control Huh‐7 cells revealed significant enrichment of the TNFA_SIGNALING_VIA_NFKB pathway in the NQO1‐overexpressing group (**Figure** **7**A). Studies have indicated that the NF‐κB signaling pathway plays a central role in the production of CXCL12, with NF‐κB dimers directly binding to the promoter region of the CXCL12 gene to initiate its transcription. Activation of the NF‐κB signaling pathway involves the phosphorylation and degradation of IκBα, and the dissociated NF‐κB dimers enter the nucleus to bind DNA and initiate gene transcription. In NQO1‐overexpressing Huh‐7 cells, we observed that the expression level of phosphorylated IκBα (p‐IκBα) were significantly increased, while the expression level of IκBα were significantly reduced (Figure 7B). This indicates activation of the NF‐κB signaling pathway. Additionally, the expression of p65 protein in the nucleus was significantly increased in NQO1‐overexpressing Huh‐7 cells (Figure 7B), further confirming the activation of the NF‐κB pathway. Moreover, in Hepa 1‐6 cells, we also observed significant upregulation of p‐IκBα and IKKα protein expression in the NQO1‐overexpressing group, with nuclear translocation of p65 (Figure 7C). This further confirmed that NQO1 promotes CXCL12 expression by activating the NF‐κB signaling pathway. These results indicate that NQO1 upregulates CXCL12 by activating the NF‐κB signaling pathway, thus promoting Treg cell recruitment and potentially accelerating the progression of HCC. To explore how NQO1 activates the NF‐κB signaling pathway, we found that NQO1‐overexpressing Huh‐7 cells were significantly enriched in the deubiquitination signaling pathway (Figure 7D). This indicates that NQO1 may activate the NF‐κB pathway by regulating protein ubiquitination. Experimental results revealed that NQO1 overexpression reduced the levels of protein ubiquitination in both Huh‐7 and Hepa 1‐6 cells (Figure 7E,F). P65 is a key subunit of NF‐κB, and its ubiquitination regulates the activation of the NF‐κB signaling pathway. By purifying NQO1 and p65 proteins, the results indicated that NQO1 can bind to p65 (Figure 7G), and the ubiquitination level of p65 was significantly reduced in the NQO1‐overexpressing group (Figure 7H,I). By contrast, the sh‐NQO1 group Hepa 1‐6 cells manifested enhanced ubiquitination of p65 (Figure 7H). This reveals that NQO1 activates the NF‐κB signaling pathway by reducing the ubiquitination of p65, which subsequently upregulates CXCL12 and promotes tumor progression. Moreover, immunoprecipitation‐mass spectrometry (IP‐MS) revealed that the NQO1 protein interacts with deubiquitinases (e.g., USP37) (Figure S9H,I), suggesting a potential role for NQO1 in modulating p65 ubiquitination through these interactions.

### 2.10 Plerixafor Enhances the Efficacy of Anti‐PD‐1 Plus Lenvatinib by Blocking CXCL12‐Mediated Treg Cell Recruitment

**Figure 8:**
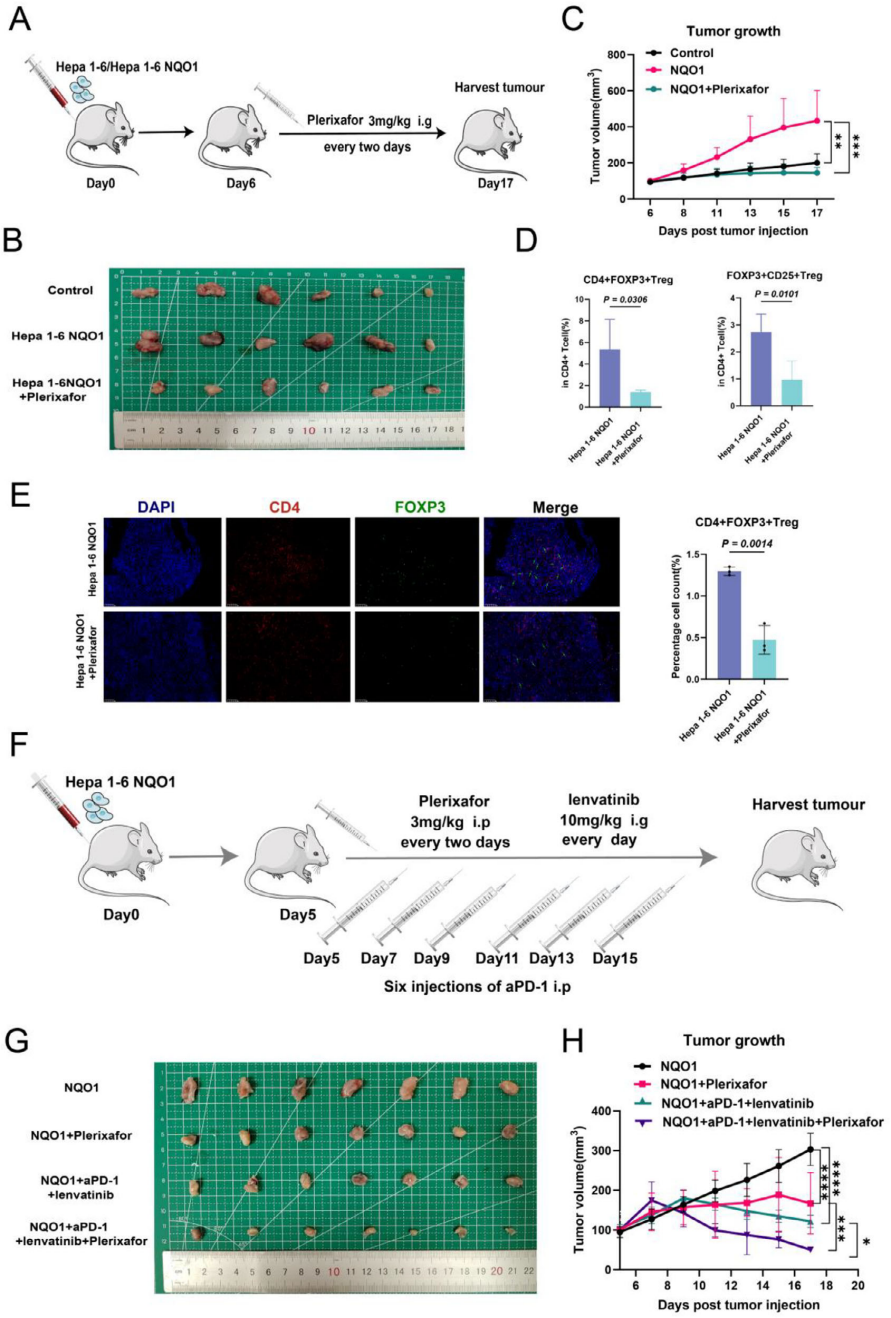
Plerixafor enhances the efficacy of anti‐PD‐1 plus lenvatinib therapy in HCC. A) Experimental flowchart showing the intraperitoneal injection of Plerixafor in mice. B) Tumor specimens from tumor‐bearing mice in different treatment groups. C) Line graph showing the changes in tumor volume over time in mice from control (*n* = 6), NQO1 (*n* = 6), and NQO1+ Plerixafor (*n* = 6) groups. D) Flow cytometry analysis showing the differences in the proportion of CD4^+^FOXP3^+^Treg and CD4^+^FOXP3^+^CD25^+^Treg cells between the two groups (Hepa 1‐6 NQO1, *n* = 5; Hepa 1‐6 NQO1+ Plerixafor, *n* = 5). E) mIHC showing the differences in CD4^+^FOXP3^+^Treg cell infiltration between the two groups (Hepa 1‐6 NQO1, *n* = 3; Hepa 1‐6 NQO1+ Plerixafor, *n* = 3). F) Flowchart showing different drug administration methods in tumor‐bearing mice. G) Tumor specimens from mice in different drug combination treatment groups. H) Line graph showing the changes in tumor volume over time in mice from NQO1 (*n* = 7), NQO1+Plerixafor (*n* = 7), NQO1+aPD‐1+lenvatin (*n* = 7), NQO1+aPD‐1+lenvatin+Plerixafor (*n* = 7). Significance in C,D,E, and H was analyzed using the two‐sided Student's *t*‐test. Data are presented as mean ±SD. Each dot corresponds to one sample. ^*^ *P* < 0.05, ^**^ *P* < 0.01, ^***^ *P* < 0.001, ^****^ *P* < 0.0001 and ns, not significant.

In vitro, Plerixafor can inhibit the CXCL12/CXCR4 axis and reduce the recruitment of Treg cells (Figure 6J). To explore the impact of Plerixafor on the TME and tumor progression in vivo, we established mouse tumor models in the control, NQO1, and NQO1 combined with Plerixafor treatment group (**Figure** **8**A). The results indicated that compared to the NQO1 group, the combined Plerixafor group exhibited significantly slower tumor growth and limited tumor volumes (Figures 8B,C; S10B, Supporting Information). This indicates that Plerixafor effectively inhibited the tumor progression promoted by NQO1.

To further investigate the effects of Plerixafor on the TME, we conducted flow cytometry analysis (Figure S10A, Supporting Information). Compared to the NQO1 group, the combined Plerixafor group indicated significantly increased infiltration of CD3^+^ T cells in the tumor tissue (Figure S10C, Supporting Information), while the infiltration of Treg cells (FOXP3^+^CD4^+^ and FOXP3^+^CD25^+^CD4^+^) was significantly reduced (Figure 8D). Additionally, mIHC further confirmed that Plerixafor effectively reduced Treg cell infiltration levels (Figure 8E). This indicates that Plerixafor may regulate the TME and inhibit tumor progression by suppressing NQO1‐mediated Treg cell infiltration.

Given that high PIVKA‐II expression tumors upregulate NQO1 to activate the NF‐κB signaling pathway, regulate CXCL12 expression, and recruit Treg cells into the TME, leading to resistance to anti‐PD‐1 plus lenvatinib. Plerixafor significantly reduces the recruitment of Treg cells, prompting investigations into its role in enhancing the efficacy of anti‐PD‐1 plus lenvatinib therapy. We established a mouse HCC model with NQO1 overexpression (Figure 8F), and the results indicated that the NQO1 + aPD‐1 + lenvatinib + Plerixafor group exhibited significantly smaller tumor volumes, with stronger effects compared to other groups (Figure 8G,H). No significant differences in body weight were observed among the different treatment groups (Figure S10D), indicating that the combination therapy is safe. Overall, Plerixafor enhances the sensitivity of HCC cells to anti‐PD‐1 plus lenvatinib therapy and may serve as an effective sensitizing agent to optimize treatment response.

## 3 Discussion

HCC is a highly heterogeneous and aggressive malignancy.^[^ ^18^, ^19^, ^20^ ^]^ Its complex TME significantly affects immunotherapy efficacy.^[^ ^21^, ^22^, ^23^ ^]^ Although PIVKA‐II is a clinically approved diagnostic biomarker for HCC diagnosis,^[^ ^24^, ^25^ ^]^ its role in tumor aggressiveness, prognosis, and response to anti‐PD‐1 plus lenvatinib therapy remains underexplored. This study addresses that gap through clinical cohort analysis and scRNA‐seq to compare the immune landscape of patients with low and high PIVKA‐II expression.

Clinically, high PIVKA‐II levels were associated with advanced BCLC stage, larger tumors, increased MVI, and shorter PFS. These features suggest that PIVKA‐II may promote HCC progression, possibly by activating the Met‐JAK‐STAT3 pathway^[^ ^15^ ^]^ and modulating angiogenic factors.^[^ ^26^ ^]^

To our knowledge, this is the first study to evaluate peripheral blood PIVKA‐II levels as a predictor of treatment response. Patients with pre‐treatment PIVKA‐II >300 mAU mL^−1^ had lower response rates, outperforming AFP and NLR as biomarkers. Post‐treatment levels of PIVKA‐II more accurately reflected therapeutic efficacy. Two mechanisms could explain this: (1) therapy reduces PIVKA‐II by inhibiting angiogenesis and tumor proliferation, and (2) responsive tumor cell apoptosis decreases PIVKA‐II release. Therefore, monitoring dynamic changes in PIVKA‐II can guide treatment adjustments.

**Figure 9:**
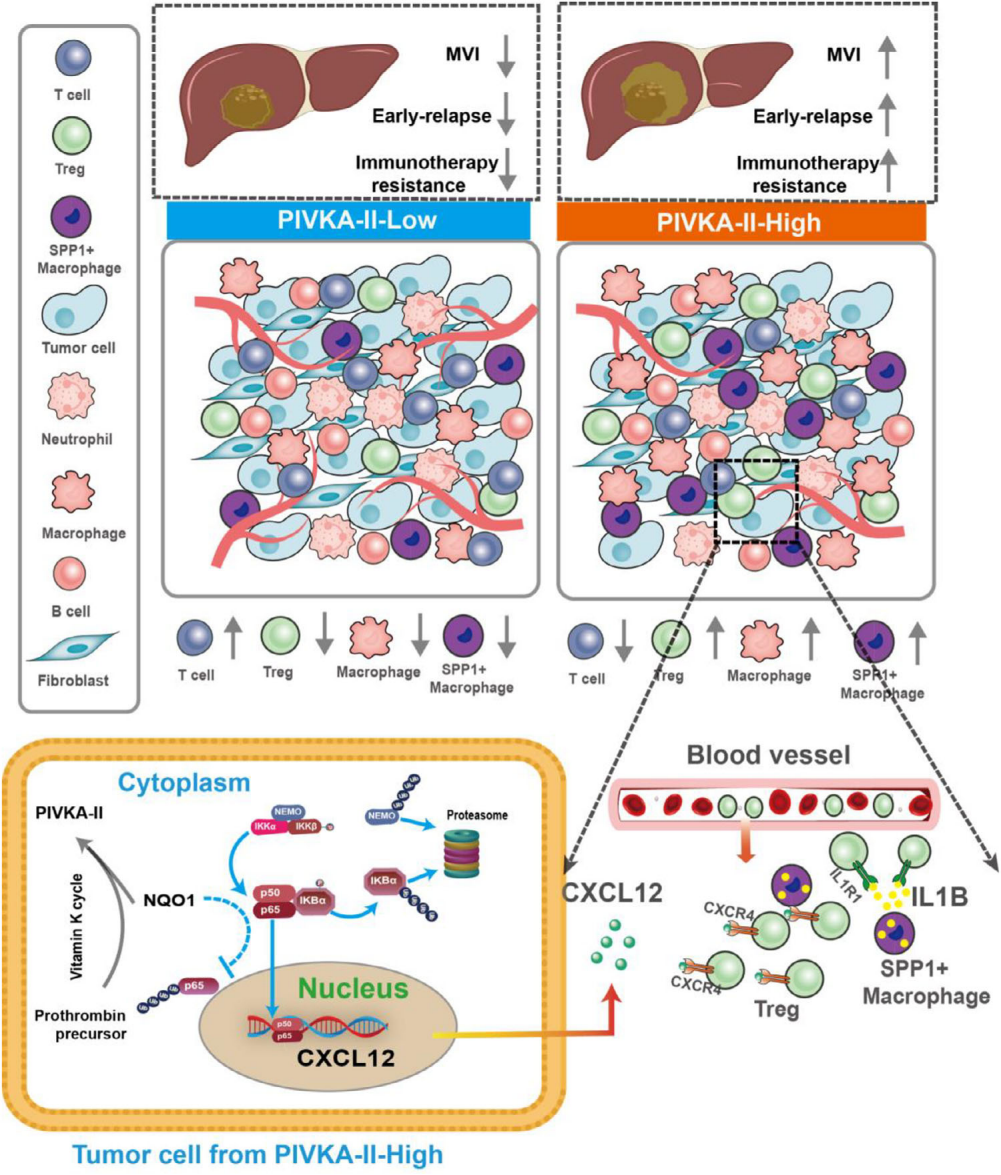
Mechanistic map of Treg cell recruitment by PIVKA‐II positive tumor cells.

At the single‐cell level, high PIVKA‐II expression was linked to reduced infiltration of cytotoxic T cells, neutrophils, and B cells, and increased immunosuppressive Tregs and SPP1^+^ TAM (**Figure** **9**). Tregs, by expressing CTLA4, TIGIT, and secreting IL‐10 and TGF‐β,^[^ ^27^, ^28^, ^29^ ^]^ suppress immune response. SPP1^+^ TAM cells promote angiogenesis and immune evasion.^[^ ^30^, ^31^ ^]^ These cells localize and interact via CXCL12‐CXCR4 and TGF‐β signaling, reinforcing immunosuppression.

In NR, immune cell heterogeneity was marked by fewer cytotoxic T and B cells and increased macrophages and Tregs. Tregs in the NR group showed transcriptional regulation by SMAD3 and STAT4, suggesting a more suppressive phenotype contributing to resistance. Gene expression analysis revealed that NQO1 was upregulated in tumors with high PIVKA‐II expression. NQO1 regulates the vitamin K cycle and promotes tumor progression via oxidative stress and metabolic reprogramming. It was significantly elevated in non‐responders, indicating a role in resistance. NQO1 may contribute by: (1) reshaping immune cell function via metabolic reprogramming;^[^ ^32^ ^]^ (2) accelerating drug metabolism or efflux;^[^ ^33^, ^34^ ^]^ and (3) enhancing tumor antioxidant capacity to escape therapy‐induced oxidative stress.^[^ ^35^ ^]^

Furthermore, NQO1 overexpression induced CXCL12 in HCC cells via NF‐κB activation. NQO1 prevents p65 ubiquitination, activating NF‐κB, which upregulates CXCL12 and recruits Tregs through CXCR4. In mouse models, NQO1 overexpression increased Tregs infiltration and worsened immunosuppression. However, the CXCR4 antagonist Plerixafor blocked this axis, reducing Tregs migration and enhancing the effect of anti‐PD‐1 plus lenvatinib. Plerixafor significantly suppressed tumor growth and improved therapy efficacy in NQO1‐overexpressing tumors. Plerixafor, a highly selective CXCR4 antagonist, has applications beyond its classical role in hematopoietic stem cell mobilization.^[^ ^36^ ^]^ By blocking the CXCL12/CXCR4 axis‐mediated tumor‐stroma interactions, it effectively disrupts the “immune‐privileged sanctuary” of tumor cells within the bone marrow/stroma and significantly remodels the immunosuppressive microenvironment.^[^ ^37^ ^]^ Preclinical and early‐phase clinical trials demonstrate that this mechanism synergistically enhances the sensitivity of solid tumors—including pancreatic cancer, glioblastoma, and colorectal cancer with liver metastases—to cytotoxic and immunotherapeutic regimens.^[^ ^38^, ^39^ ^]^ Reported adverse events are predominantly transient and reversible, comprising Grade 1–2 injection‐site reactions, mild gastrointestinal discomfort, or known combination therapy‐related myelosuppression and immune‐related events, collectively supporting a favorable tolerability profile.

These findings position NQO1 as a central mediator of immune suppression and therapy resistance via the CXCL12‐CXCR4 axis. Targeting this pathway could overcome resistance in high PIVKA‐II expression HCC. As a pivotal biomarker, serial monitoring of PIVKA‐II effectively discriminates HCC subtypes and enables prognostic stratification. Clinical practice should establish individualized treatment pathways guided by PIVKA‐II thresholds, prompting timely therapeutic adjustments combined with local interventions to enhance efficacy. This stratified management strategy carries significant clinical implications for improving patient survival outcomes.

## 4 Conclusion

Peripheral blood PIVKA‐II levels are a promising predictor of response to anti‐PD‐1 plus lenvatinib in HCC. High PIVKA‐II is associated with an immunosuppressive TME characterized by increased Treg cell infiltration, contributing to treatment resistance. NQO1 promotes CXCL12 expression via NF‐κB activation, further enhancing Tregs recruitment. Blocking the CXCL12‐CXCR4 axis with Plerixafor significantly improved therapeutic efficacy, offering a promising strategy to overcome resistance in patients with high PIVKA‐II expression.

## 5 Experimental Section

### Patient Clinical Data and Tissue Samples

This study included patients treated at the Department of Hepatobiliary and Pancreatic Surgery, Chinese PLA General Hospital, between January 2019 and June 2024. All patients provided written informed consent and had complete clinical data. A total of 156 HCC patients who underwent surgical resection were enrolled; none had received prior treatments such as radiofrequency ablation or immunotherapy. Preoperative PIVKA‐II data were available for all. Additionally, data were collected from 104 HCC patients who received anti‐PD‐1 plus lenvatinib, including pre‐treatment PIVKA‐II data for 86 patients. Treatment response was assessed per RECIST 1.1 criteria.

For scRNA‐seq, tumor, adjacent tissue, and peripheral blood samples were collected from 22 patients with available PIVKA‐II values: 15 underwent first‐stage surgical resection, and 7 received anti‐PD‐1 plus lenvatinib therapy prior to surgery. For validation, tumor tissue from 10 additional HCC patients treated with anti‐PD‐1 plus lenvatinib was subjected to bulk RNA sequencing. Furthermore, paired tumor and adjacent non‐tumor tissue from 17 HCC patients who underwent surgical resection were collected for bulk RNA sequencing to validate the findings.

### Mice

C57BL/6J mice (male,4–6 weeks old, ≈20g) were purchased and housed under SPF conditions (12‐h light/dark cycle) with ad libitum access to food and water. Mice were acclimated for at least 7 days and individually identified with an ear tag.

All animal experiments conducted in this study were reviewed and approved by the Ethics Committee of the First Medical Center of the Chinese PLA General Hospital (Approval No. 2020‐X16‐96).

### Mouse Models

Hepa1‐6 cells were trypsinized, centrifuged, and resuspended in PBS with Matrigel. Under isoflurane anesthetized, 100 µL of the cell suspension was injected subcutaneously into the shared left lower abdomen using a 0.5ml syringe. The injection site was gently compressed to prevent leakage. Tumor length and width, and body weight were recorded from day 3 post‐injection. On days 5–6, mice were stratified by tumor size and randomized to receive Plerixafor (3mg kg^−1^, every other day), anti‐PD1 antibody (5mg kg^−1^, every other day), or lenvatinib (5mg kg^−1^, daily via gavage). Tumor volume was calculated as (length × width^2^) / 2 and measured every two days together with body weight. At the experimental endpoint, mice were euthanized by isoflurane. Blood was collected for plasma analysis, and tumors were excised, photographed, and processed for further analysis and flow cytometry. The remaining tumor tissue was fixed in neutral‐buffered formalin.

### Cell Lines and Culture Methods

Huh‐7 and Hepa 1‐6 cells were obtained from the American Type Culture Collection (ATCC) or the Cell Bank of the Chinese Academy of Sciences (CCAS, China). Cells were cultured in DMEM supplemented with 10% (v/v) fetal bovine serum (FBS) and 1% (v/v) penicillin−streptomycin at 37 °C in a humidified 5% CO2 incubator.

### Cell Migration and Invasion Assays

For invasion assays, Matrigel was diluted 1:8 with serum‐free medium, and 60 µL was added to the Transwell insert; gels polymerized at 37 °C for 3h and were then hydrated with 100 µL serum‐free medium for 30 min. Cells were detached with 0.25% trypsin for 2‐3 min, centrifuged, and resuspended in serum‐free medium at 10⁵ cells/mL. The lower chamber contained 500 µL DMEM with 10% serum. The Transwell insert was placed carefully into the well, and 200 µL of cell suspension was added to the upper chamber. After 48 h of incubation, inserts were washed with PBS. Cells in the upper chamber were removed, and the membrane was fixed with 600 µL of 4% paraformaldehyde for 30 min. After washing with PBS, the membrane was stained with 0.1% crystal violet for 30 min, rinsed with water, and cleaned with a cotton swab.

### Western Blot

Proteins were mixed with 5× loading buffer and heated at 95 °C for 10 min. For gel preparation, 5 mL each of lower gel A and B solutions were combined with 30 µL polymerizing agent, poured into the casting plate, and left to polymerize for 10 min. Similarly, 1 mL each of upper gel A and B solutions was mixed with 12 µL polymerizing agent, poured on top of the lower gel, and fitted with a comb. After preparing the 1 × electrophoresis buffer, 10 µL of protein marker, and 10 µL of each sample were loaded. Electrophoresis was run at 80V through the stacking gel and 120V through the resolving gel. Proteins were transferred to a methanol‐activated PVDF membrane at 4 °C. Membranes were blocked in 10% non‐fat milk in TBST for 60 min at room temperature and incubated overnight at 4 °C with gentle agitation. After TBST washes, membranes were incubated with secondary antibodies for 60 min at room temperature. Following additional washes, the ECL substrate was applied, and protein bands were visualized.

### ELISA

Cell culture supernatants were collected and centrifuged at 1000 rpm for 20 min, then stored at −80 °C or used immediately. Mouse blood was collected in EDTA or heparin tubes, centrifuged at 1000 rpm for 15 min at 4 °C, and stored at −8 °C. ELISA kits were performed per the manufacturer's instructions. Standards, samples, and controls (100 µL/well, run in duplicate) were incubated at 37 °C for 60 min. Biotinylated antibody (100 µL) was added and incubated for another 60 min. Wells were washed three times with 300 µL wash solution. Enzyme conjugate (100 µL) was added and incubated for 30 min, followed by five washes. Substrate solution (90 µL) was added and incubated for 15 min, and the reaction was stopped with 50 µL of stop solution. OD values were measured at 450 nm. Standard curves were generated using OD (*X*‐axis) and standard concentration (*Y*‐axis) on a log‐log scale.

### Fresh Tissue Dissociation into Single Cells

After resection, tissue were rinsed in cold PBS, placed in tissue‐preservation solution, and processed within 4 h. After trimming to remove clots, fat, and debris, tissue were minced into 1–3 mm^3^ fragments, and digested at 37 °C for 30 min in collagenase‐based enzyme, followed by filtration through a 70 µm strainer and centrifugation. Red blood cells were lysed using ACK buffer when necessary. Cells were resuspended in DPBS and assessed with AO/PI staining. Preparations meeting ≥85% viability, total cell count ≥1 ×10^5^, debris <15%, and cell diameter compatible with the single‐cell platform were used for library construction.

### scRNA‐Seq Data Preprocessing

Raw BCL files were converted to FASTQ using Illumina bcl2fastq. Reads were aligned to the human genome (GRCh38) with Cell Ranger with the STAR algorithm. Unique molecular identifiers (UMIs) were used to quantify gene expression. Data were analyzed using Seurat (R). Cells with <200 detected genes, >8000 genes, <300 UMIs, or >10% mitochondrial reads were excluded. Data were merged, normalized, and scaled. The top 2000 variable genes were selected for PCA. Batch effects were corrected with the Harmony algorithm. Uniform Manifold Approximation and Projection (UMAP) was utilized for visualization. Clustering and cell‐type annotation were performed using canonical marker genes and Azimuth references.

### GSEA Enrichment Analysis

Gene Set Enrichment Analysis (GSEA) was performed using the GSEA software and MSigDB version 7.4. Genes were ranked by log2 fold change with FDR q ≤ 0.25 were considered significant. Results were visualized in R using ggplot2.

### Cell Communication Analysis

NicheNet analysis was used to identify ligands (average Log2FC > 1). The top 20% of ligands were analyzed for ligand‐receptor interactions. Results were visualized with ggplot2. Cell Chat was also applied to identify overexpressed ligands and receptors in specific subpopulations. Communication probabilities were computed and visualized using netVisual_bubble() and the circlize package.

### External Independent Datasets

To validate the single‐cell data findings, several external datasets were utilized. The Cancer Genome Atlas (TCGA) provided bulk RNA sequencing data from 365 HCC patients. GSE14520 offered tumor tissue data from 219 patients. scRNA‐seq data from 8 HCC patients treated with anti‐PD‐1 plus lenvatinib (GSE235863) were also analyzed. Bulk RNA‐seq data from 15 treated patients (4 non‐responders, 11 responders) were included from GSE235863. Additionally, data from GSE202069 (17 patients; 9 non‐responders, 8 responders) and GSE279750 (10 patients; 4 non‐responders, 6 responders) were analyzed.

### Statistical Analysis

Statistical analyses were performed using R (4.1.2) and GraphPad Prism (V.8.0). Continuous variables were summarized as mean ± standard deviation, while non‐normal continuous variables were reported as median (interquartile range, IQR). Categorical variables were described using frequencies (n) and percentages (%). Survival analysis was performed using Kaplan‐Meier curves, and Log‐rank tests were applied for group comparisons. To compare two independent samples, we applied t‐tests for normal variables, the Wilcoxon test for non‐normal variables, and Chi‐square or Fisher's exact tests for categorical variables. A P‐value of <0.05 was considered statistically significant.

### Ethical Approval and Consent to Participate

Liver tumor tissue were obtained from the First Medical Center of the Chinese PLA General Hospital. All patients signed an informed consent to participate in the study before donating the samples. The study was reviewed and approved by the Ethics Committee of the First Medical Center of the Chinese PLA General Hospital(S2018‐111‐01). The study was conducted in accordance with the principles of the Declaration of Helsinki.

## Conflict of Interest

The authors declare no conflict of interest.

## Authors' Contribution

B.G., Y.W., Z.S., H.T., and Y.C. performed the data analysis and wrote the manuscript. These authors contributed equally to this work. Y.C., H.J., W.Z., B.H., Z.L., G.M., X.L., J.L., Y.X., Y.X., T.W., and B.L. participated in the collection of specimens. X.Z., S.J., and C.L. reviewed and revised the manuscript. SL played a guiding role in the overall study. These corresponding authors contributed equally to this work. All authors contributed to the article and approved the submitted version.

## Supporting information



Supporting Information

Supporting Information

Supporting Information

Supporting Information

Supporting Information

Supporting Information

Supporting Information

Supporting Information

Supporting Information

Supporting Information

Supporting Information

Supporting Information

Supporting Information

## Data Availability

Data are available upon reasonable request. All data generated that are relevant to the results presented in this article are included in this article. Other data that were irrelevant for the results presented herein are available from the corresponding author, SL, upon reasonable request.
