# Supplementary material for: NQO1/p65/CXCL12 Axis‐Recruited Tregs Mediate Resistance to Anti‐PD‐1 Plus Lenvatinib Therapy in PIVKA‐II‐Positive Hepatocellular Carcinoma
Source: Adv Sci (Weinh). 2025 Sep 30;12(46):e11152. doi: 10.1002/advs.202511152 (PMC12697844; doi:10.1002/advs.202511152)
Supplement: Supplementary file 1 — Supporting Information [file ADVS-12-e11152-s009.docx]

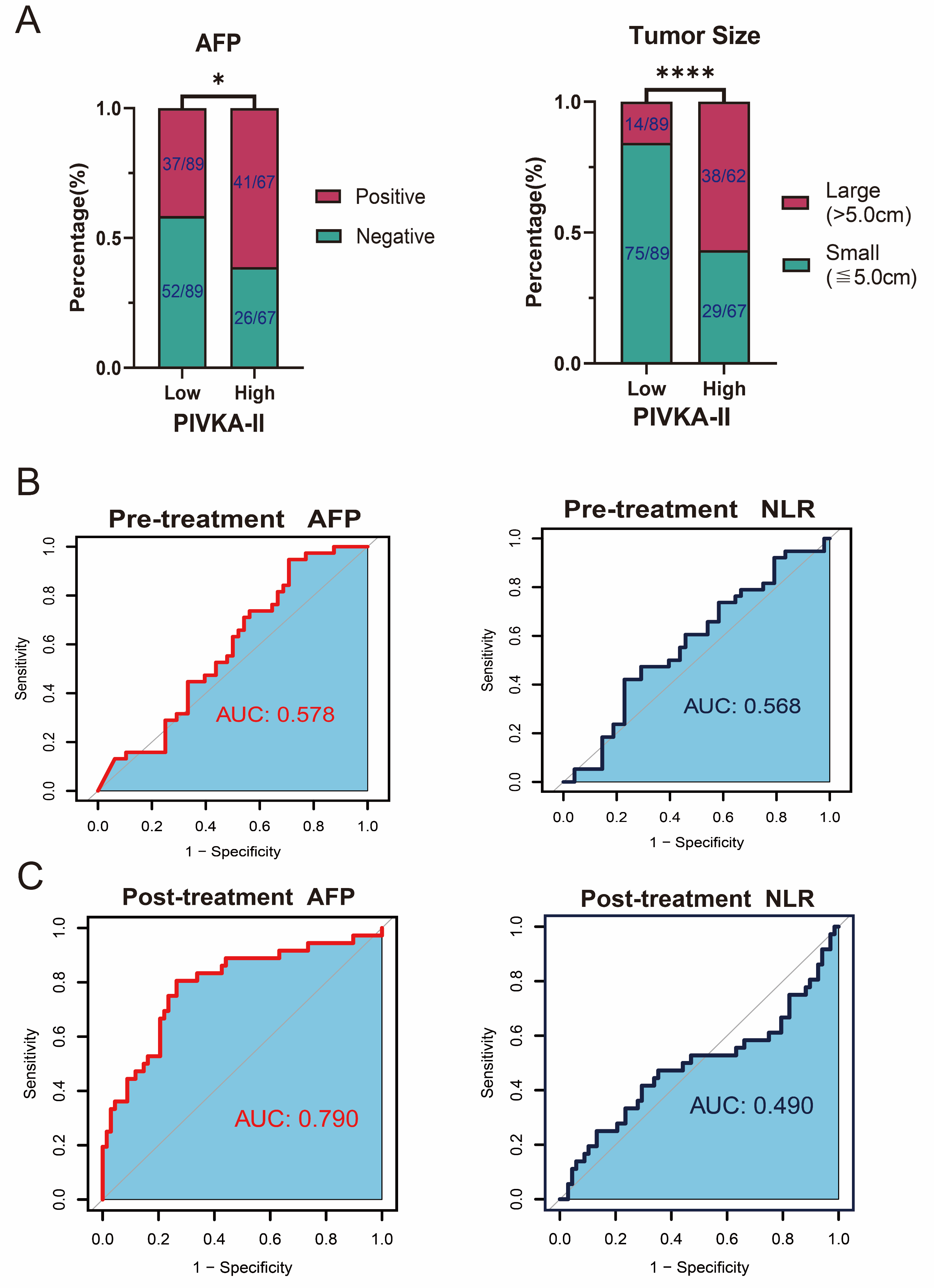


**Figure S1** Prediction of treatment response by AFP and NLR pre- and post-treatment.A) Bar chart showing the relationship between high and low PIVKA-II expression groups, AFP levels, and tumor diameter. B) ROC curve showing the predictive efficacy of pre-treatment AFP and NLR levels in peripheral blood for treatment response.C) ROC curve showing the predictive efficacy of post-treatment PIVKA-II levels in peripheral blood for treatment response. Significance in A and B was analyzed using the Chi-square test. ^*^*P* < 0.05, ^****^*P* < 0.0001 and ns, not significant.


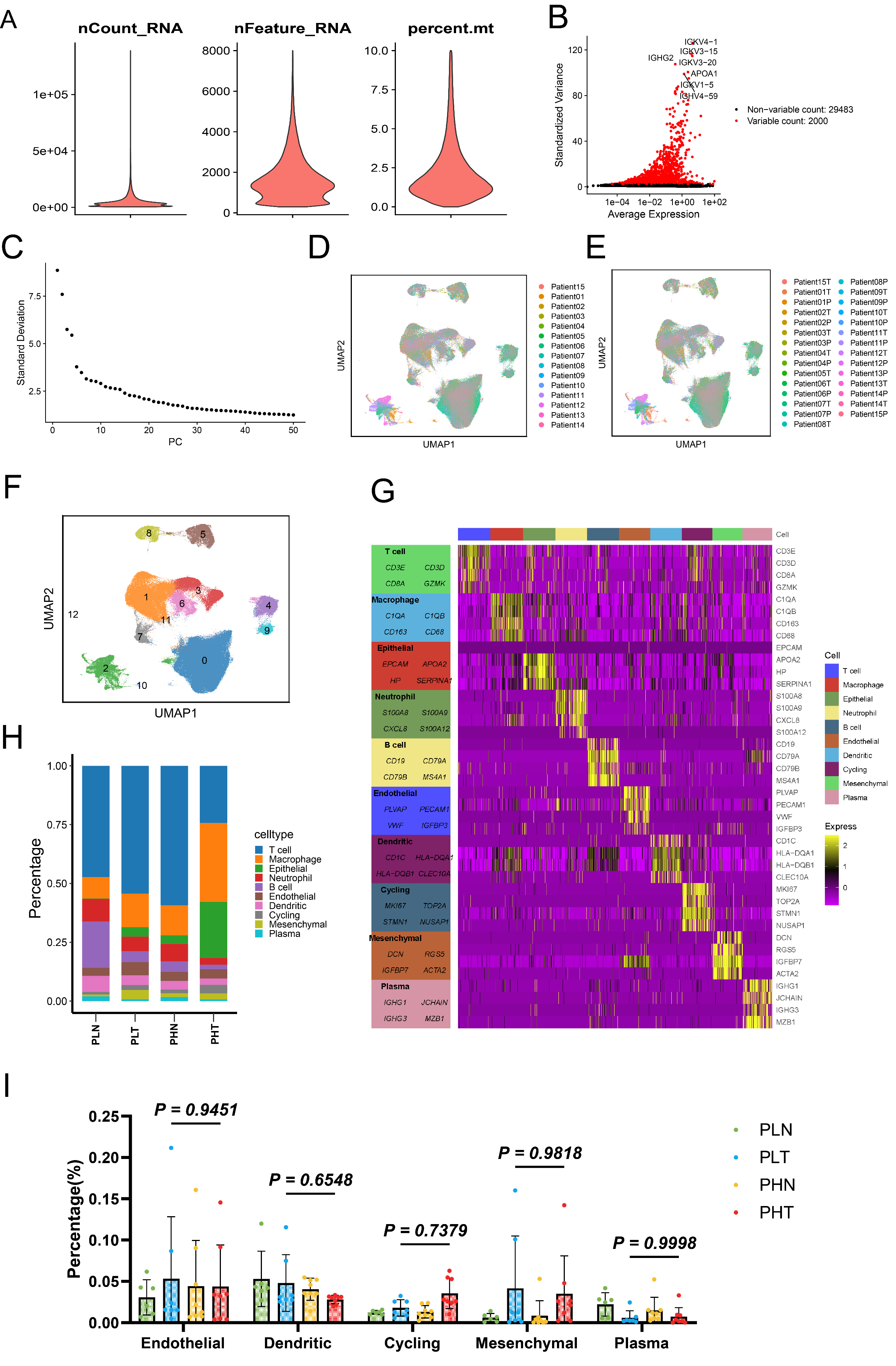


**Figure S2** Preprocessing of scRNA-seq data from 15 primary HCC patients. A) Violin plot showing the filtering criteria for single-cell data.B) Dot plot showing the top 2000 highly variable genes.C) Elbow plot showing the first 50 principal components.D) UMAP plot showing the distribution of cells from different patients.E) UMAP plot showing the distribution of cells from tumor and adjacent normal tissues from different patients.F) UMAP plot showing the distribution of different cell clusters.G) Heatmap showing the expression of marker genes in different cell types.H) Stacked bar chart showing the proportion of different cells in PLT, PLN, PHT, and PHN.I) Bar chart showing the differences in the proportions of endothelial cells, dendritic cells, cycling cells, stromal cells, and plasma cells among the PLT, PLN, PHT, and PHN groups. Significance in I was analyzed using the two-sided Student’s t-test.Data are presented as mean±SD. Each dot corresponds to one sample.


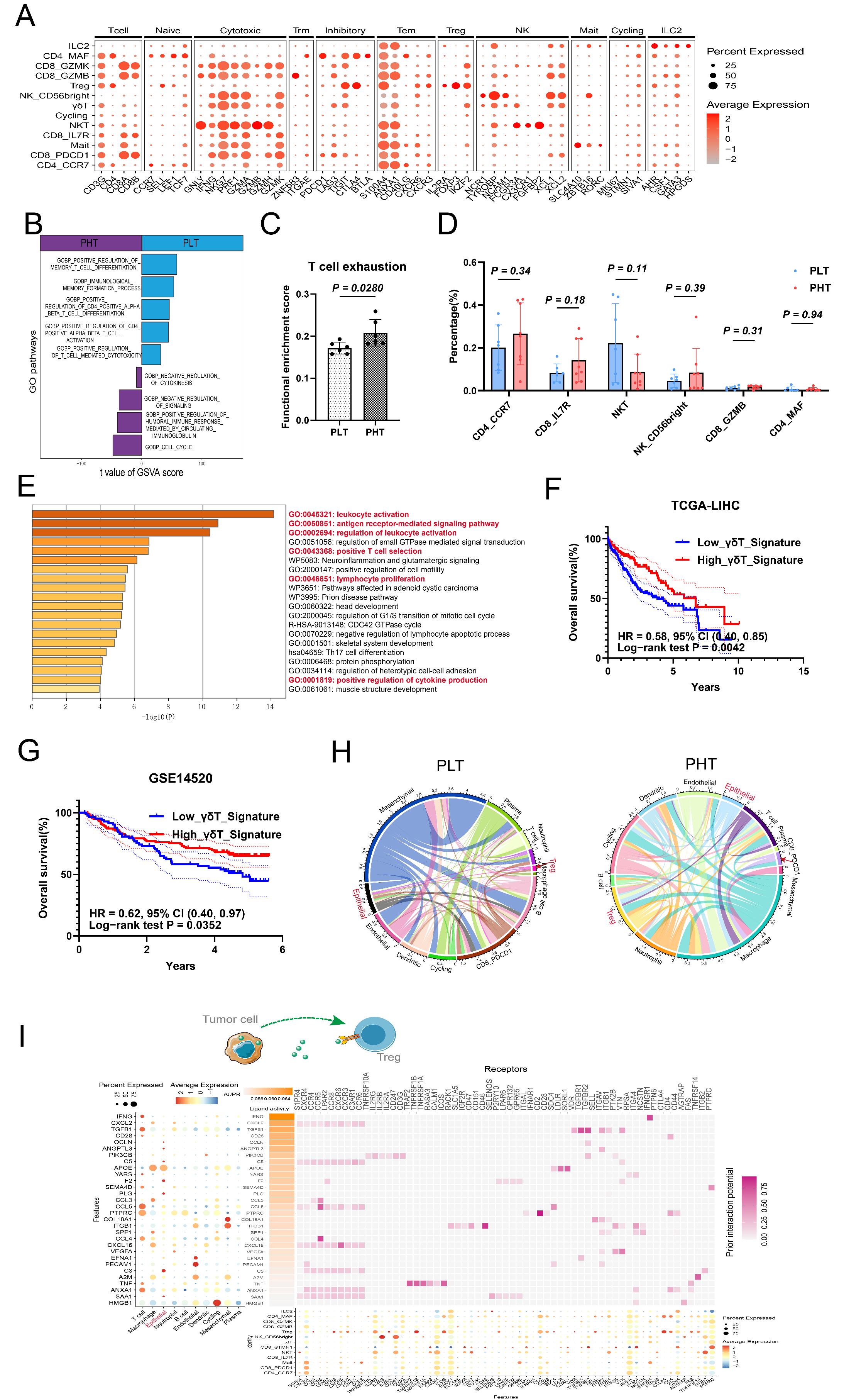


**Figure S3** Enhanced interactions between tumor cells and Treg cells in the high PIVKA-II expression group. A) Bubble chart showing the overexpressed characteristic genes in T cell subgroups.

B) Bar chart showing the differences in GSVA enrichment pathways between the two groups.C) Bar chart showing the differences in T cell exhaustion scores between the two groups (PLT, n=7; PHT, n=8). D) Bar chart showing the differences in the proportions of T and NK cell subgroups between PLT(n=7) and PHT(n=8). E) Bar chart showing the signaling pathways enriched by upregulated genes in γδT cells.F) Kaplan-Meier survival curve showing the relationship between γδT characteristic scores and OS in the TCGA dataset. *P* value was calculated using the log-rank test.G) Kaplan-Meier survival curve showing the relationship between γδT characteristic scores and OS in the GSE14520 dataset. *P* value was calculated using the log-rank test.H) Chord diagram showing the interaction strength between different cell types in PHT and PLT.I) Integrated heatmap and bubble chart showing ligand-receptor interactions between tumor cells and Treg cells. Left chart: Bubble chart displaying the expression of ligand-related genes in different cell types.Top right chart: Heatmap showing ligand-receptor interactions between tumor cells and Treg cells, arranged by ligand activity.Bottom right chart: Bubble chart showing the expression of receptor-related genes in T cell subgroups. Significance in C and D was analyzed using the two-sided Student’s t-test.Data are presented as mean±SD. Each dot corresponds to one sample.
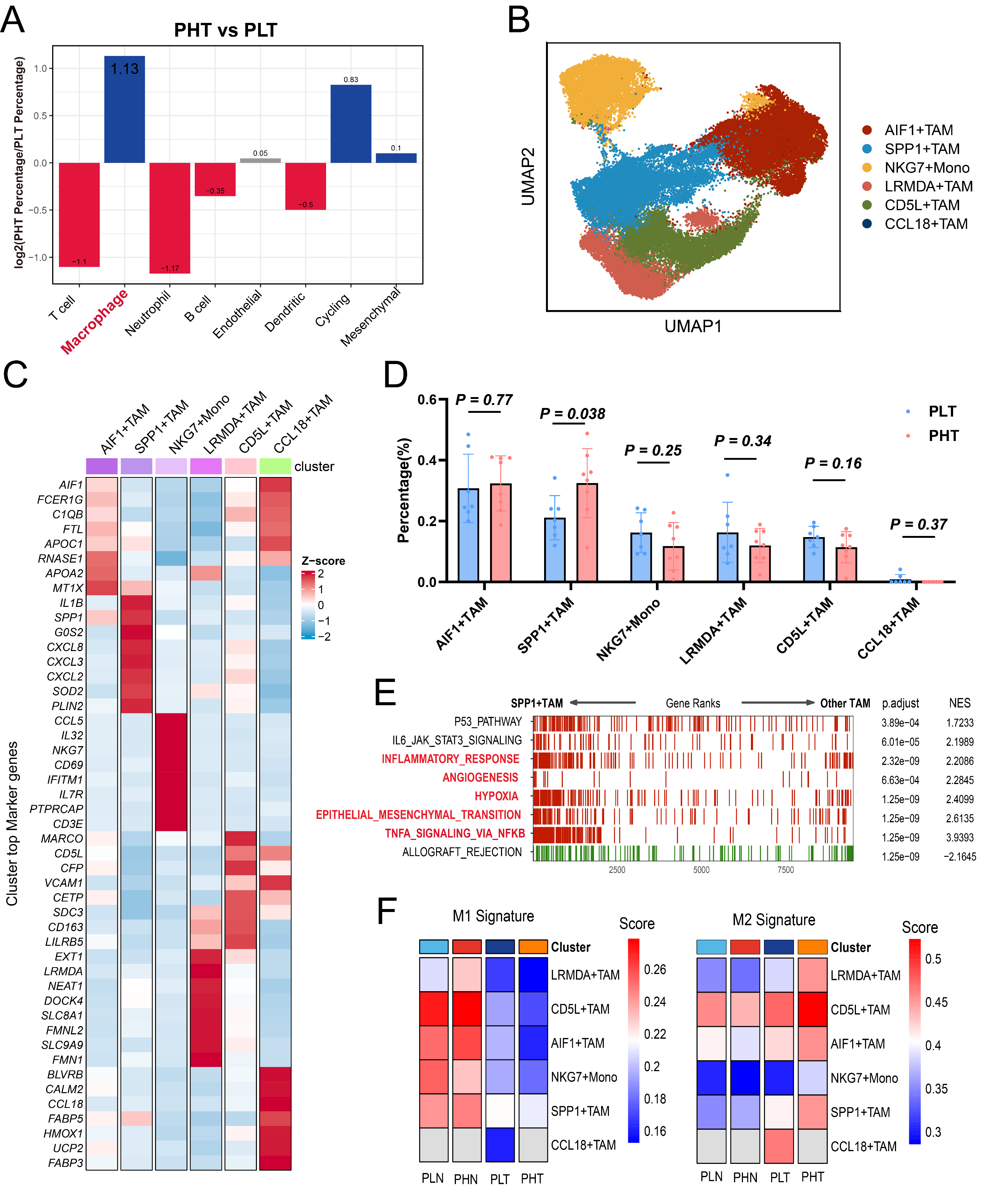


**Figure S4** Increased infiltration of SPP1^+^ TAMs in the TME of patients with high PIVKA-II expression.A) Bar chart showing the fold differences in the infiltration ratios of different cell types between PHT and PLT.B) UMAP plot showing the distribution of macrophage subgroups.C) Heatmap showing the overexpressed characteristic genes in different macrophage subgroups.D) Bar chart showing the differences in the proportion of macrophage subgroups between PLT(n=7) and PHT(n=8). E) Heatmap showing the signaling pathways involved in SPP1^+^ TAMs.F) Heatmap showing M1 and M2 polarization scores for macrophage subgroups across different groups. Significance in D was analyzed using the two-sided Student’s t-test.Data are presented as mean±SD. Each dot corresponds to one sample.
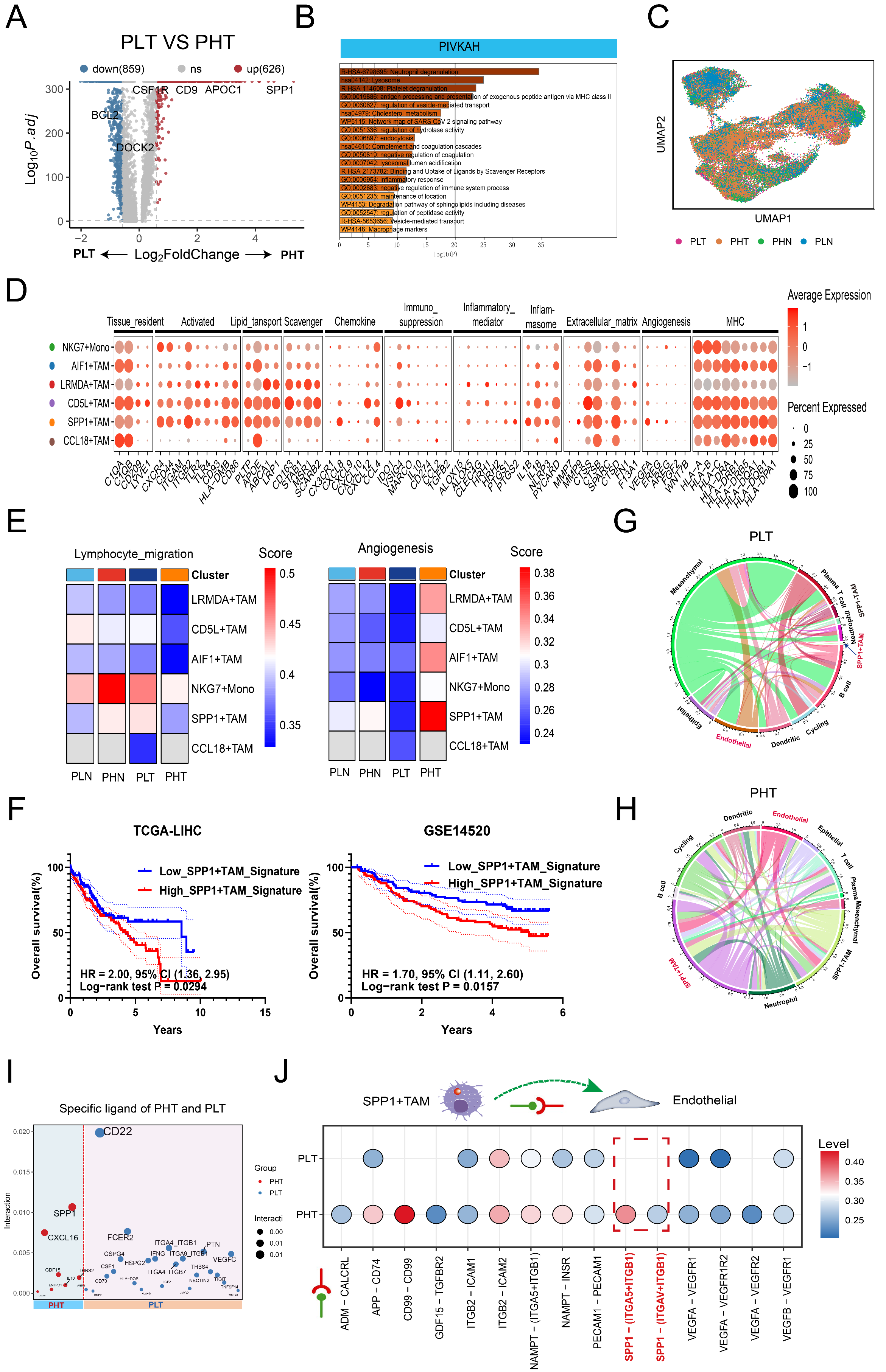


**Figure S5** Specific interactions between SPP1⁺ TAMs and endothelial cells in the TME of high PIVKA-II expression patients promote angiogenesis. A) Volcano plot showing differentially expressed genes between PHT and PLT.B) Bar chart showing the signaling pathways involved in the upregulated genes in PHT.C) UMAP plot showing the distribution of macrophages derived from PLN, PLT, PHN, and PHT.D) Bubble chart showing the overexpressed functionally related genes in different macrophage subgroups.E) Heatmap showing the functional scores of macrophage subgroups in different groups.F) Kaplan-Meier survival curves showing the relationship between SPP1⁺ TAM signature scores and OS in the TCGA and GSE14520 datasets. *P* value was calculated using the log-rank test.G) Chord diagram showing the interaction strength between SPP1^+^ TAMs and different cells in PLT.H) Chord diagram showing the interaction strength between SPP1^+^ TAMs and different cells in PHT.I) Scatter plot showing the ligands specifically enriched in PHT and PLT.J) Bubble chart showing the interaction strength between SPP1^+^ TAMs and endothelial cells.
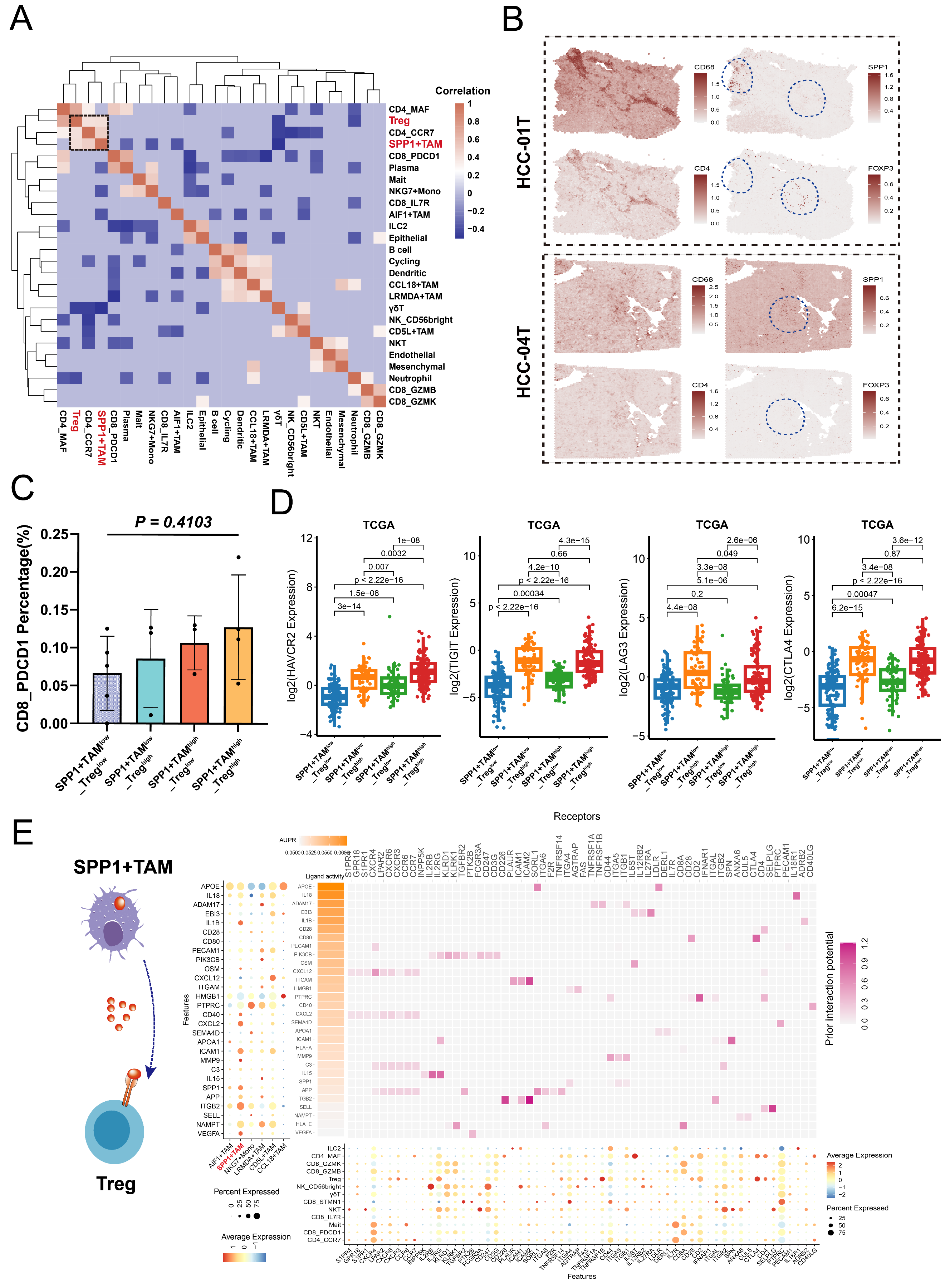


**Figure S6** SPP1⁺ TAMs interact with Treg cells in the high PIVKA-II expression group to promote an immunosuppressive microenvironment. A) Heatmap showing similar infiltration patterns of SPP1^+^ TAMs and Treg cells in the TME.B) Enhanced spatial feature map showing the expression of CD68, SPP1, CD4, and FOXP3 in HCC tumor tissues.C) Bar chart showing the proportion of CD8_PDCD1 cells across different groups(SPP1^+^TAM^low^_Treg^low^,n=4; SPP1^+^TAM^low^_Treg^high^,n=3; SPP1^+^TAM^high^_Treg^low^,n=3;SPP1^+^TAM^high^_Treg^high^,n=4). D) Boxplot showing the expression of different immune checkpoint-related genes in different groups(SPP1^+^TAM^low^_Treg^low^,n=127; SPP1^+^TAM^low^_Treg^high^,n=55; SPP1^+^TAM^high^_Treg^low^,n=55;SPP1^+^TAM^high^_Treg^high^,n=128) in the TCGA datasets. E) Integrated heatmap and bubble chart showing ligand-receptor interactions between SPP1⁺ TAMs and Treg cells. Left chart: Bubble chart showing the expression of ligand-related genes in different types of macrophage cells.Top right chart: Heatmap showing the ligand-receptor interactions between SPP1^+^ TAMs and Treg cells, arranged by ligand activity.Bottom right chart: Bubble chart showing the expression of receptor-related genes in T cell subgroups. Significance in C was analyzed using the two-sided Student’s t-test.Data are presented as mean±SD. Each dot corresponds to one sample.Significance in D was analyzed using the two-sided Wilcoxon rank-sum test.Center line: median; box edges: 25th/75th percentiles; whiskers: 1.5*IQR; upper and lower bars: 95% CI.


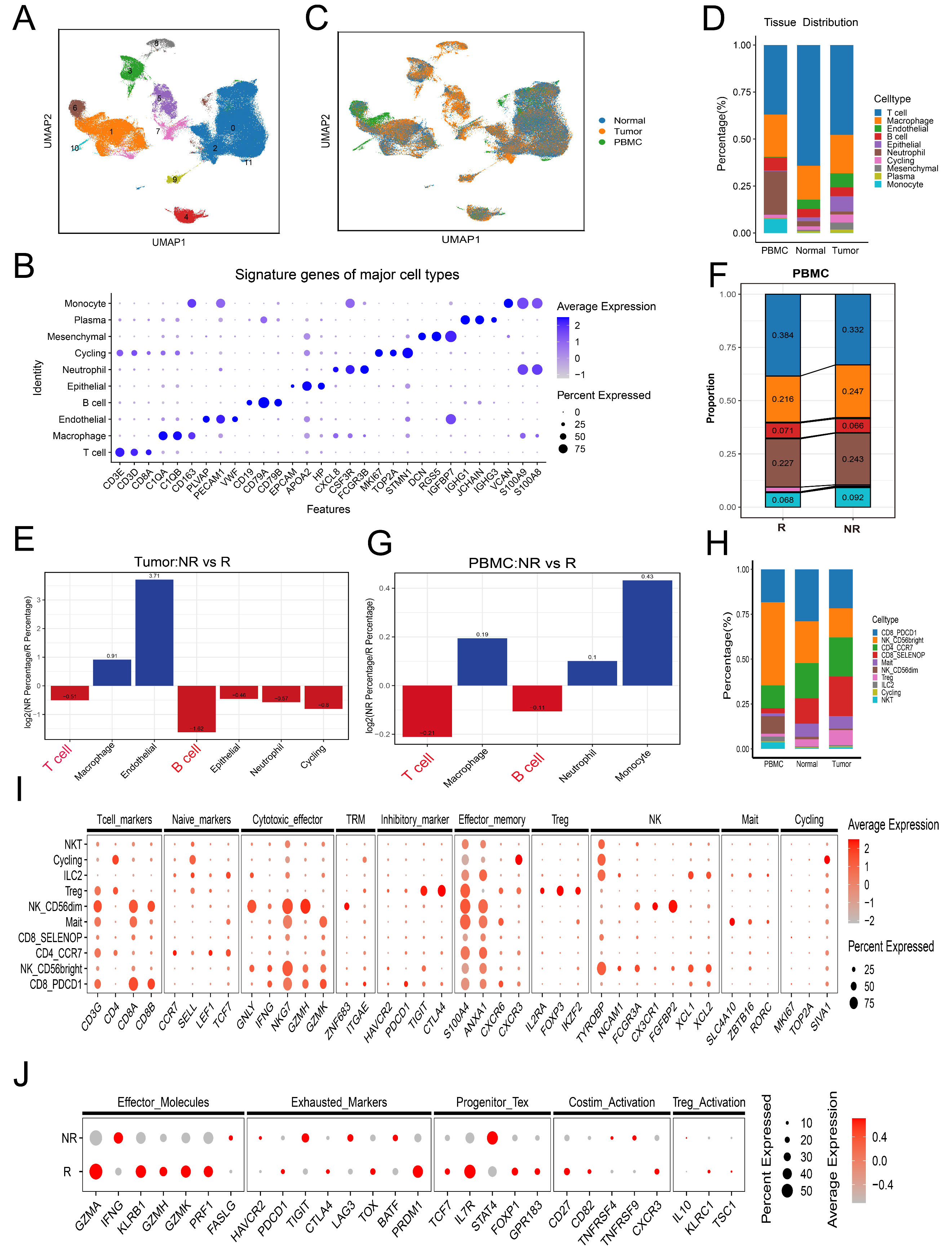


**Figure S7** Impact of the immunosuppressive microenvironment in high PIVKA-II expression patients on response to anti-PD-1 plus lenvatinib therapy. A) UMAP plot showing the distribution of cell clusters.B) Bubble chart showing the overexpressed characteristic genes in different cell types.C) UMAP plot showing the distribution of cells derived from normal tissues, peripheral blood, and tumor tissues.D) Stacked bar chart showing the cell composition from different tissue sources.E) Bar chart showing the fold differences in the infiltration ratios of different cell types between PHT and PLT in tumor tissues.F) Stacked bar chart showing the differences in cell composition between PHT and PLT in peripheral blood.G) Bar chart showing the fold differences in the infiltration ratios of different cell types in peripheral blood between PHT and PLT.H) Stacked bar chart showing the composition of T cell subgroups in different tissues.I) Bubble chart showing the expression of characteristic genes in different T cell subgroups.J) Bubble chart showing differential expression of T cell function-related genes between NR and R groups. NR: non-responder;R: responder.
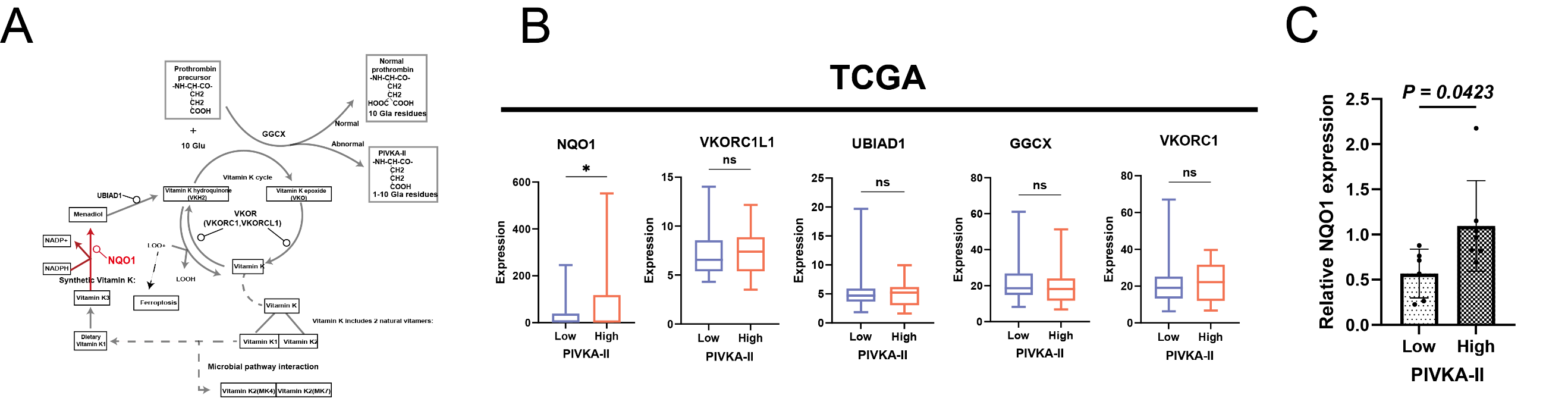


**Figure S8** Upregulation of NQO1 in tumor cells from the high PIVKA-II expression group.A) The connection between the vitamin K cycle pathway and PIVKA-II formation.B) Box plots showing the expression of key vitamin K cycle pathway genes in high (n=23) vs. low (n=68) PIVKA-II expression groups from the TCGA dataset. Center line: median; box edges: 25th/75th percentiles; whiskers: 1.5*IQR; upper and lower bars: 95% CI; statistical analysis was performed using the two-sided Wilcoxon rank-sum test. ^*^*P* < 0.05 and ns, not significant.C) Bar graph showing NQO1 protein levels in the two groups. Data are presented as mean±SD. Each dot corresponds to one sample. Statistical significance was performed using a two-sided Student’s t-test.
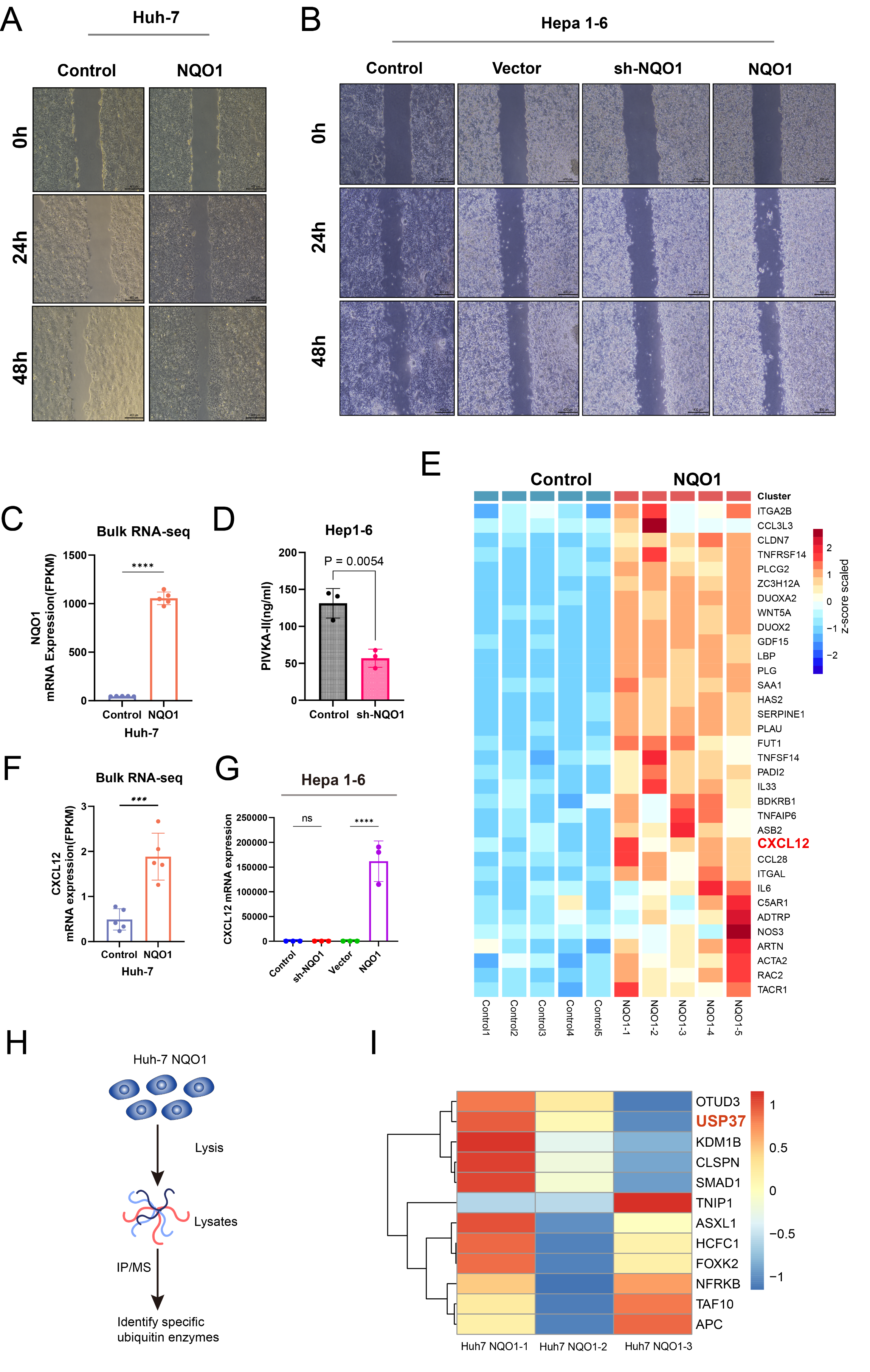


**Figure S9** NQO1 promotes migration and invasion of HCC cells.

A) Wound healing assay showing the differences in migration ability between control and NQO1-overexpressing group Huh-7 cells.B) Wound healing assay showing the differences in migration ability between Control, Vector, sh-NQO1, and NQO1 groups Hepa 1-6 cells.C) Bar chart showing the expression of NQO1 between NQO1-overexpressing(n=5) and control group(n=5) Huh-7 cells based on bulk RNA sequencing data. D) Bar chart showing the difference in the expression level of PIVKA-II in Hepa 1-6 cells of different treatment condition groups(Control,n=3;sh-NQO1,n=3). E) Heatmap showing the expression differences of lymphocyte migration-related genes between NQO1-overexpressing and control group Huh-7 cells.

F) Bar chart showing the expression of CXCL12 between NQO1-overexpressing(n=5) and control group(n=5) Huh-7 cells based on bulk RNA sequencing data. G) qPCR results showing CXCL12 mRNA expression in Hepa 1-6 cells from the Control (n=3), Vector (n=3), sh-NQO1 (n=3), and NQO1 (n=3) groups.H) Schematic diagram of the integrated proteomics and IP-MS analysis used to identify NQO1 interaction partners. I) Heatmap showing dysregulated proteins (red, upregulated proteins; blue, downregulated proteins) identified via proteomics assays. Significance in C,D,F, and J was analyzed using the two-sided Student’s t-test.Data are presented as mean±SD. Each dot corresponds to one sample.


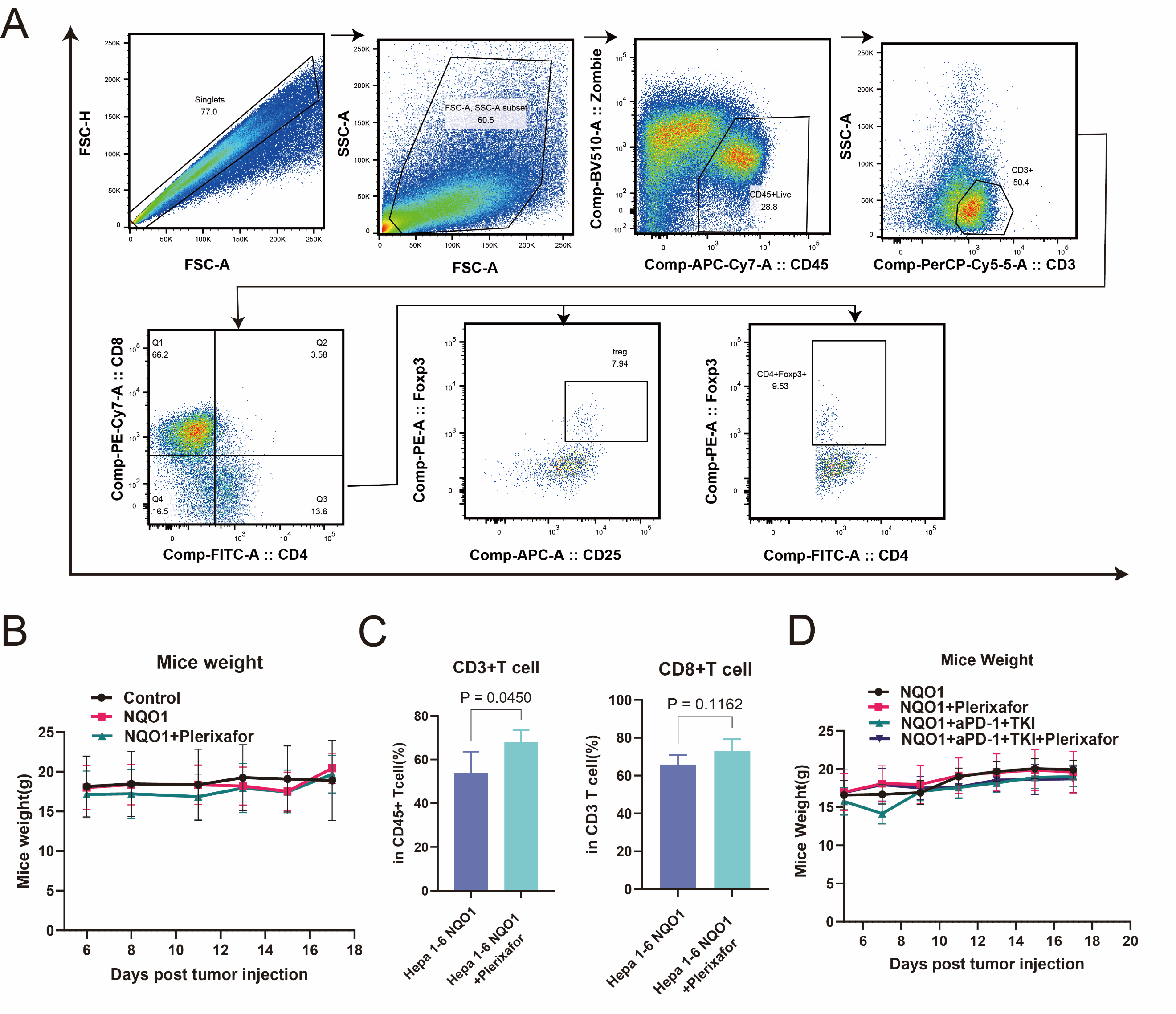


**Fig. S10** Plerixafor enhances T cell infiltration in tumors.A) Representative flow cytometry plots showing the gating strategy for T cells isolated from tumors.B) Line graph showing the changes in body weight over time in mice from different groups.C) Flow cytometry analysis showing the proportions of CD3⁺ T cells and CD8⁺ T cells in the two groups(Hepa 1-6 NQO1,n=5; Hepa 1-6 NQO1+ Plerixafor,n=5). D) Line graph showing the changes in body weight over time in mice from different drug treatment groups. Significance in C was analyzed using the two-sided Student’s t-test.Data are presented as mean±SD.
